# Interaction between the mitochondrial adaptor MIRO and the motor adaptor TRAK

**DOI:** 10.1016/j.jbc.2023.105441

**Published:** 2023-11-08

**Authors:** Elana E. Baltrusaitis, Erika E. Ravitch, Adam R. Fenton, Tania A. Perez, Erika L.F. Holzbaur, Roberto Dominguez

**Affiliations:** 1 Department of Physiology, Perelman School of Medicine, University of Pennsylvania, Philadelphia, Pennsylvania, USA; 2 Biochemistry and Molecular Biophysics Graduate Group, Perelman School of Medicine, University of Pennsylvania, Philadelphia, Pennsylvania, USA; 3 Cell and Molecular Biology Graduate Group, Perelman School of Medicine, University of Pennsylvania, Philadelphia, USA

**Keywords:** mitochondrial dynamics, motor adaptor, calcium, EF-hand, GTPase, mutagenesis, isothermal titration calorimetry (ITC)

## Abstract

MIRO (mitochondrial Rho GTPase) consists of two GTPase domains flanking two Ca^2+^-binding EF-hand domains. A C-terminal transmembrane helix anchors MIRO to the outer mitochondrial membrane, where it functions as a general adaptor for the recruitment of cytoskeletal proteins that control mitochondrial dynamics. One protein recruited by MIRO is TRAK (trafficking kinesin-binding protein), which in turn recruits the microtubule-based motors kinesin-1 and dynein-dynactin. The mechanism by which MIRO interacts with TRAK is not well understood. Here, we map and quantitatively characterize the interaction of human MIRO1 and TRAK1 and test its potential regulation by Ca^2+^ and/or GTP binding. TRAK1 binds MIRO1 with low micromolar affinity. The interaction was mapped to a fragment comprising MIRO1’s EF-hands and C-terminal GTPase domain and to a conserved sequence motif within TRAK1 residues 394 to 431, immediately C-terminal to the Spindly motif. This sequence is sufficient for MIRO1 binding *in vitro* and is necessary for MIRO1-dependent localization of TRAK1 to mitochondria in cells. MIRO1’s EF-hands bind Ca^2+^ with dissociation constants (*K*_*D*_) of 3.9 μM and 300 nM. This suggests that under cellular conditions one EF-hand may be constitutively bound to Ca^2+^ whereas the other EF-hand binds Ca^2+^ in a regulated manner, depending on its local concentration. Yet, the MIRO1-TRAK1 interaction is independent of Ca^2+^ binding to the EF-hands and of the nucleotide state (GDP or GTP) of the C-terminal GTPase. The interaction is also independent of TRAK1 dimerization, such that a TRAK1 dimer can be expected to bind two MIRO1 molecules on the mitochondrial surface.

Mitochondrial Rho GTPase (MIRO, isoforms 1 and 2; 60% sequence identity) acts as a general Ca^2+^- and GTP-regulated adaptor on the outer mitochondrial membrane. MIRO is implicated in mitochondrial transport, morphogenesis, inheritance, homoeostasis, degradation, and contacts with the endoplasmic reticulum (1, 2, 3, 4, 5, 6, 7, 8). MIRO consists of N- and C-terminal GTPase domains (nGTPase, residues 1–168 and cGTPase, residues 416–579; numbering is for human MIRO1) that surround two pairs of EF-hands. Each EF-hand pair consists of a canonical Ca^2+^-binding EF-hand and a “hidden” EF-hand that does not bind Ca^2+^ (9), and hosts in its hydrophobic pocket a ligand-mimic helix (10). Together, an EF-hand pair with its bound ligand-mimic helix is referred to as the ELM domain (ELM1, residues 183–274; ELM2, residues 303–384) (10) (Fig. 1*A*). Additionally, MIRO has a C-terminal transmembrane helix (residues 590–618) that inserts into the outer mitochondrial membrane, where MIRO is predominantly localized (1).

**Figure 1:**
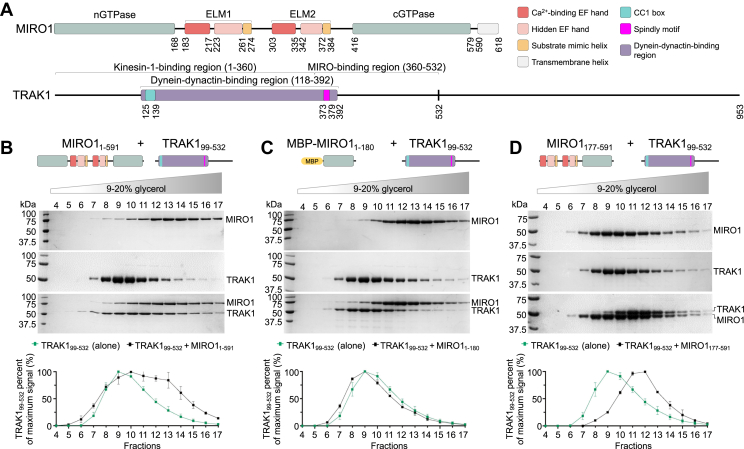
**MIRO1 binds TRAK1 through a fragment comprising the EF-hands and cGTPase.***A*, domain diagrams of MIRO1 and TRAK1 (nGTPase and cGTPase, N- and C-terminal GTPase domains; ELM 1 and 2, EF-hand pair ligand mimic one and two; CC1 box, coiled-coil one box). Each ELM domain consists of a Ca^2+^-binding EF-hand (*red*), a hidden EF-hand that does not bind Ca^2+^ (*pink*), and a ligand mimic helix (*orange*). By analogy with other dynein-dynactin adaptors (21, 22), the dynein-dynactin-binding region of TRAK1 can be mapped to residues 118 to 392 (*purple*) and comprises the CC1 box (*cyan*) and the Spindly motif (*magenta*). The binding sites of kinesin-1 and MIRO1 have been approximately mapped to residues 1 to 360 (12, 14, 16, 17, 18) and 360 to 532 (17), respectively. *B*–*D*, migration of MIRO1_1-591_, MBP-MIRO1_1-180_, MIRO1_177-591_ alone and together with TRAK1_99-532_ in a 5 to 30% glycerol gradient. The figures show representative SDS-PAGE analyses of the fractions 4 to 17 (9–20% glycerol) containing these proteins. Densitometric analysis of the gels is shown at the *bottom*. For each fraction, the average TRAK1 signal (alone, *green* or with MIRO1 constructs, *black*) from N = 3 independent experiments (Fig. S2) is reported as the percentage of the average signal for the TRAK1 fraction with the maximum intensity. MBP, maltose-binding protein; MIRO, mitochondrial Rho GTPase; TRAK, trafficking kinesin-binding protein.

One of the proteins MIRO recruits to mitochondria is trafficking kinesin protein (TRAK, isoforms 1 and 2; 49% sequence identity), which in turn recruits the microtubule-based motors kinesin-1 and dynein-dynactin (11). Sequence identity among TRAK isoforms is somewhat higher (53%) for the N-terminal ∼400-amino acid region, which harbors the dynein-dynactin and kinesin-1-binding sites (12, 13, 14, 15, 16, 17, 18). TRAK belongs to a family of activating adaptors that, while generally unrelated, share long regions of coiled-coil and are capable of recruiting and activating dynein and dynactin for long-range processive motility (19, 20). By analogy with known cryo-EM structures of other activating adaptors in complex with dynein and dynactin (21, 22), the region of TRAK involved in binding dynein-dynactin consists of amino acids 118 to 392 (numbering is for human TRAK1). This region is predicted to form a long coiled-coil along the F-actin-like dynactin minifilament, which forms the core of the dynactin complex, and includes the CC1-box (residues 125–139) and Spindly (residues 373–379) motifs at its N- and C-terminal ends, respectively. The CC1-box interacts with the dynein light intermediate chain, and this interaction is important for processive dynein motility (23, 24, 25, 26). While the binding site of kinesin-1 has not been precisely mapped, several studies converge on a fragment comprising TRAK residues 1 to 360 (12, 13, 14, 15, 17, 18). Thus, the binding sites of kinesin-1 and dynein-dynactin may overlap. Yet, both motors have been localized to a single TRAK adaptor, which suggests the existence of regulatory mechanisms that avoid a futile tug-of-war between the two motors during the mitochondrial transport toward the opposite ends of the microtubule (16, 17). Moreover, knockdown or inhibition of either motor impairs mitochondrial motility in both microtubule directions, suggesting functional interdependence of the two motor systems (14, 27).

TRAK (11, 14, 28) and MIRO (29, 30, 31, 32, 33) are both essential for mitochondrial motility and distribution in neurons. KO studies in mice identify MIRO1, and not MIRO2, as the main isoform associated with mitochondrial transport in axons and dendrites (33). Similarly, TRAK1 appears to play a predominant role compared to TRAK2 in mitochondrial motility in mature neurons (11). Perhaps for this reason, most studies have focused on isoforms MIRO1 and TRAK1. Yet, given a relatively high sequence identity among the isoforms of each protein, our underlying hypothesis is that they interact through a conserved mechanism, which nevertheless remains poorly understood due in part to conflicting evidence in the literature.

Some studies have implicated MIRO’s nGTPase in the interaction with TRAK, while specifically excluding the EF-hands and cGTPase (2, 32, 34, 35). These studies, however, disagree as to whether the nucleotide state of the nGTPase (GTP or GDP) is important (32, 34, 35) or not (2) for this interaction. On the other hand, elevated local Ca^2+^ concentrations arrest mitochondrial trafficking in different cell types (36, 37, 38), and this effect is suppressed by mutations in the EF-hands of MIRO1 (4, 30, 39, 40). While these studies appear to directly implicate the EF-hands in interactions with either kinesin-1 or TRAK1 (4, 30, 39), another study suggests that the effect of the EF-hand mutations is indirect, by reducing Ca^2+^ entry into mitochondria, which in turn acts as a signal to control mitochondrial motility (40). A recent study finds that MIRO1 and TRAK1/2 interact and comigrate in the absence or the presence of Ca^2+^ (17). It is therefore unclear which MIRO domain(s) bind TRAK and whether the interaction is regulated by Ca^2+^ and/or nucleotide.

Here, we map and quantitatively characterize the interaction of MIRO1 with TRAK1 and explore its potential regulation by Ca^2+^ and the nucleotide state (GTP or GDP) of MIRO1. MIRO1 binds TRAK1 through a fragment comprising the EF-hands and cGTPase. The interaction is independent of Ca^2+^ binding to the EF-hands and is unaffected by the nucleotide state of the cGTPase. On TRAK1, the interaction was mapped to a conserved motif (residues 394–431) C terminal to the Spindly motif. This sequence is necessary for MIRO1-dependent localization of TRAK1 to mitochondria in cells. The interaction is independent of TRAK1 dimerization, such that a TRAK1 dimer can be expected to bind to two MIRO1 molecules on the mitochondrial membrane.

## Results

### MIRO1 binds TRAK1 through a fragment comprising the EF-hands and cGTPase

Using pull-down experiments, a recent study found that MIRO1 forms a quaternary complex with kinesin-1 (KIF5B), dynein-dynactin, and a TRAK1 fragment comprising residues 1 to 532 (TRAK1_1-532_), but not with a shorter fragment consisting of residues 1 to 360 (TRAK1_1-360_) (17). These results suggested that a MIRO1-binding site is located within TRAK1 residues 360 to 532. Moreover, these authors found that the association and comigration of MIRO1 with TRAK1_1-532_, dynein-dynactin, and KIF5B was independent of the nucleotide state or the presence of Ca^2+^. Based on these results, we designed MIRO1 and TRAK1 constructs to specifically map their interaction using glycerol gradient cosedimentation. Experiments were initially performed in the presence of 1 mM CaCl_2_, 1 mM MgCl_2_, 0.1 μM GTP. The longest TRAK1 construct analyzed consisted of residues 99 to 532 (TRAK1_99-532_), comprising the previously identified MIRO1-binding region (17), but avoiding the less conserved N-terminal sequence that precedes the coiled-coil that binds dynein-dynactin (Figs. 1*A* and S1). Crystal structures have been determined of human MIRO1’s nGTPase (41) and a fragment comprising the EF-hands and cGTPase (42). These structures and AlphaFold (43) suggest that the nGTPase is detached from the EF-hands and cGTPase, with the latter two being more tightly packed against one another. These considerations informed the design of three MIRO1 constructs: MIRO1_1-591_, a near full-length construct lacking the C-terminal transmembrane helix that reduces solubility; maltose-binding protein (MBP)-MIRO1_1-180_, corresponding to MIRO1’s nGTPase and fused to MBP for enhanced purity and solubility; and MIRO1_177-591_, comprising the EF-hands and cGTPase.

MIRO1_1-591_ comigrated with TRAK1_99-532_ in the glycerol gradient, as indicated by a rightward (higher mass) shift of the main peak of TRAK1_99-532_ when ran together with MIRO1_1-591_ as compared to control (TRAK1_99-532_ ran alone) (Figs. 1*B* and S2*A*). The migration profile of TRAK1_99-532_ did not change with or without MBP-MIRO1_1-180_ (Figs. 1*C* and S2*B*). This result suggests a lack of interaction and disagrees with previous reports that mapped this interaction to the nGTPase using cellular pull-downs and point mutations in the catalytic site of the nGTPase (2, 32, 34, 35). In contrast, TRAK1_99-532_ comigrated with MIRO1_177-591_, suggesting that the TRAK1-binding site is contained within the EF-hands and/or cGTPase (Figs. 1*D* and S2*C*).

In the sedimentation assays, MIRO1_1-591_ and TRAK1_99-532_ migrated over a broad range of glycerol concentrations, both alone and together (Fig. 1, *A*–*C*), which suggests these proteins are extended, assume multiple conformations, or form higher molecular weight oligomers. Mass photometry was used to explore the oligomerization state of the three MIRO1 constructs used above, which by SDS-PAGE migrate as single bands (Fig. S3*A*). Of note, this method uses low protein concentrations (∼20–150 nM), such that weakly interacting species tend to dissociate. Moreover, mass measurement become less accurate for proteins with molecular weights <100 kDa (44). It is nevertheless a useful technique to study large proteins and protein complexes or to assess sample homogeneity (45), which is our purpose here. With MIRO1_1-591_, we observed multiple species, and the distribution profile was very similar in the presence of 100 μM CaCl_2_ or 1 mM EGTA, which chelates Ca^2+^ (Fig. S3*B*). This result indicates that MIRO1_1-591_ tends to oligomerize and that Ca^2+^ binding to the EF-hands is unlikely to produce a substantial conformational change. MBP-MIRO1_1-180_, which does not include the EF-hands, was only analyzed in 1 mM EGTA and also showed multiple species (Fig. S3*C*). Therefore, the nGTPase may be at least in part responsible for MIRO1’s tendency to oligomerize, consistent with structural studies showing that the nGTPase forms dimers (41). In contrast, at this low concentration (150 nM) MIRO1_177-591_ displayed a single peak, both in the presence or the absence of Ca^2+^, suggesting a rather stable conformation independent of Ca^2+^ binding (Fig. S3*D*). This result is also consistent with crystal structures of this fragment showing fundamentally the same conformation independent of the bound cation or nucleotide (10, 42). MIRO1_177-591_ is thus used in most experiments below, as it comprises the TRAK1-binding site (Fig. 1*D*) and appears homogeneous (Fig. S3*D*).

With TRAK1_99-532_, we observed two peaks (Fig. S3*E*), with measured masses 66 ± 4.8 kDa and 91 ± 9 kDa (theoretical mass of monomer: 53.5 kDa). While these mass measurements are not accurate, this result suggests that by itself and at low concentration (125 nM) the TRAK1 coiled-coil dimer is unstable, and the protein may exist in monomer-dimer equilibrium. The coiled-coil is likely stabilized in the complex with dynein-dynactin, as observed in the cryo-EM structures of other adaptors (21, 22).

### TRAK1 binds MIRO1 through a conserved sequence C terminal of the Spindly motif

**Figure 2:**
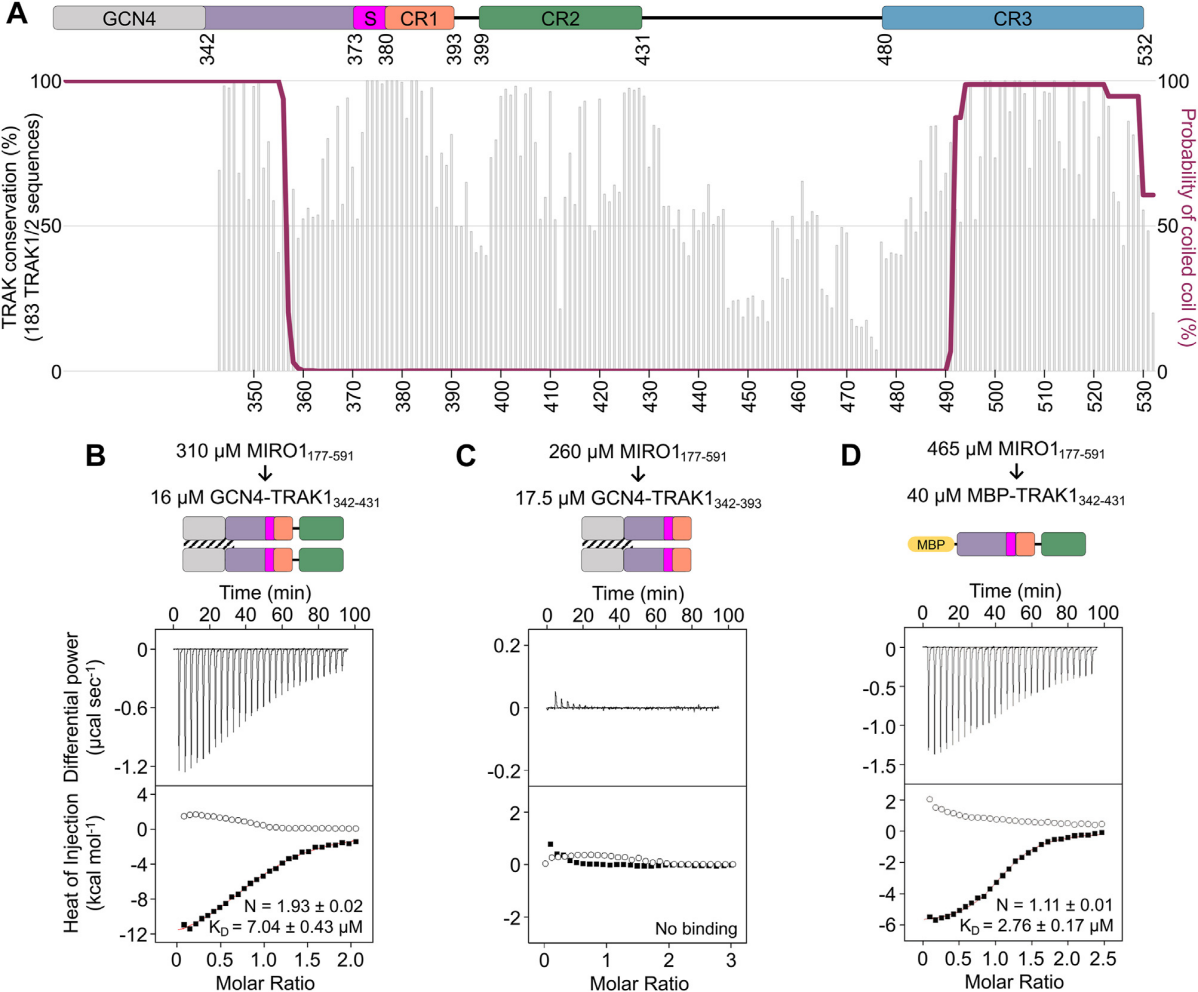
**TRAK1 binds MIRO1 through a conserved sequence C-terminal of the Spindly motif**. *A*, domain diagram (*top*), sequence conservation (*bottom*, *gray* *bars*), and coiled-coil prediction (*bottom*, *maroon trace*) of construct GCN4-TRAK1_342-431_ (also Fig. S1). Per-residue conservation scores were calculated with the program Scorecons (64) from an alignment of 183 vertebrate TRAK sequences, including 93 TRAK1 and 90 TRAK2 sequences. The coiled-coil prediction used a 28-amino acid window. Three conserved regions (CR1, CR2, and CR3) identified C terminally to the Spindly motif are highlighted. *B*–*D*, representative ITC titrations of MIRO1_177-591_ into the indicated TRAK1 constructs from N = 3 independent experiments (Table S1). Prior to each titration, the proteins in the syringe (*top*) and in the cell (*bottom*) were codialyzed for 3 days in ITC buffer with 50 μM CaCl_2_. Listed with each titration are the concentrations of the protein in the syringe and in the cell and, for interacting proteins, the parameters of the fit (stoichiometry N and dissociation constant *K*_*D*_) to a binding isotherm (*red line*). Errors correspond to the SD of the fits. *Open symbols* correspond to control titrations into buffer. ITC, isothermal titration calorimetry; MIRO, mitochondrial Rho GTPase; TRAK, trafficking kinesin-binding protein.

Having identified the region of MIRO1 implicated in TRAK1 binding, we set out to map the binding site on TRAK1. As mentioned above, the MIRO1-binding site is expected to be located within TRAK1 residues 360 to 532 (17), which is also consistent with our cosedimentation analysis (Fig. 1, *B* and *D*). We focused on this area and designed TRAK1 constructs taking into consideration sequence conservation and coiled-coil prediction (Fig. 2*A*), two factors which may be important for the interaction with MIRO1. Because the coiled-coil prediction drops around residue 352 (Fig. S1), the TRAK1 constructs analyzed here start with residue E342, within the region of high coiled-coil probability. To further stabilize the coiled-coil, a 30-amino acid GCN4 leucine-zipper (46) was added N terminally to E342, ensuring that the heptad repeats of the leucine-zipper and TRAK1 matched at the point of fusion (Fig. S4).

The MIRO-TRAK interaction is likely conserved among TRAK species and isoforms and should therefore involve a region of high sequence conservation. Other than the Spindly motif, three regions of high sequence conservation can be identified within TRAK1 residues 360 to 532 (Fig. 2*A*): CR1 (380–393), CR2 (399–431), and CR3 (480–532). Isothermal titration calorimetry (ITC) was used to quantitatively test the interaction of MIRO1_177-591_ with TRAK1 constructs lacking either CR3 (GCN4-TRAK1_342-431_) or CR2 and CR3 (GCN4-TRAK1_342-393_) (Fig. S5). Experiments were performed in 50 μM CaCl_2_, 1 mM MgCl_2_, 0.1 μM GTP (higher Ca^2+^ concentrations precipitated the GCN4 hybrid constructs). MIRO1_177-591_ bound GCN4-TRAK1_342-431_ with *K*_*D*_ = 7.04 μM and stoichiometry N = 1.93 (Fig. 2*B* and Table S1) but did not bind GCN4-TRAK1_342-393_ (Fig. 2*C* and Table S1). This finding suggests the MIRO1 binding site lies within the 33-residue CR2 region. MIRO1_177-591_ also bound the monomeric construct MBP-TRAK1_342-431_, in which GCN4 was replaced with MBP, with similar affinity (*K*_*D*_ = 2.76 μM) as for GCN4-TRAK1_342-431_ but with stoichiometry N = 1.11 (Fig. 2*D* and Table S1). This result suggests that a TRAK1 dimer can potentially bind to two MIRO1 molecules on the mitochondrial surface.

In these ITC experiments, the control titrations of MIRO1_177-591_ into buffer (open symbols) displayed a small amount of dissociation heat (endothermic), which was subtracted from the heats of binding to TRAK1 constructs (exothermic). Dissociation is not observed when monomeric TRAK1 constructs are injected into MIRO1_177-591_ (see below).

### The MIRO1-TRAK1 interaction is independent of Ca^2+^ binding to the EF-hand domains

**Figure 3:**
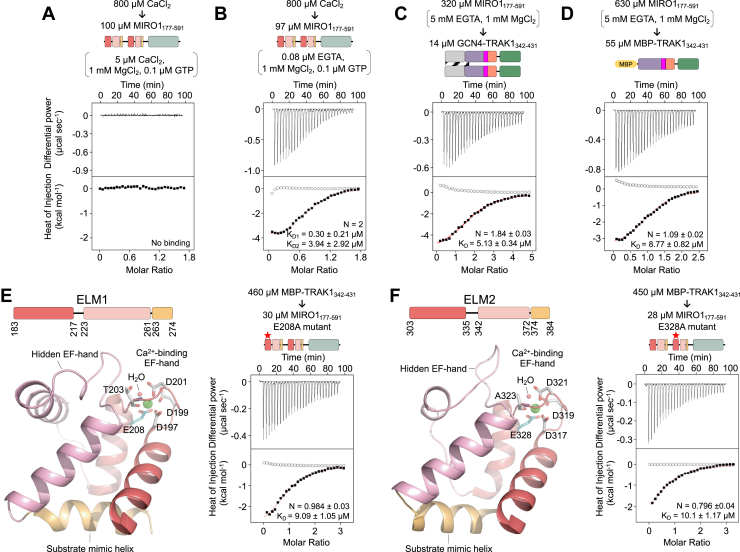
**The MIRO1-TRAK1 interaction is independent of Ca**^**2+**^**binding to the EF-hand domains**. *A* and *B*, representative ITC titrations (see also Table S1) of 800 μM CaCl_2_ into MIRO1_177-591_. Prior to the titrations, MIRO1_177-591_ was dialyzed for 3 days in ITC buffer with either 5 μM CaCl_2_ (*A*) or 5 mM EGTA followed by stepwise removal of EGTA to a final concentration of 0.08 μM (*B*). The titrated CaCl_2_ was dissolved in the dialysis buffer. *C* and *D*, representative ITC titrations from N = 3 independent experiments (Table S1) of MIRO1_177-591_ into the indicated TRAK1 constructs. Proteins were dialyzed in ITC buffer with 5 mM EGTA. *E* and *F*, representative ITC titrations from N = 3 independent experiments (Table S1) of MBP-TRAK1_342-431_ into MIRO1_177-591_ mutants E208A (*E*) and E328A (*F*). Proteins were dialyzed in ITC buffer with 5 mM EGTA. Ribbon diagrams (*left*, color coded as in Figure 1*A*, Ca^2+^ ions in *green*) highlight the location of the mutated amino acid (*cyan* Cα) within the Ca^2+^-binding loop of each ELM domain (42). Shown with each ITC titration in this figure are the concentrations of the protein (or CaCl_2_) in the syringe and in the cell and the parameters of the fit (stoichiometry N and dissociation constant *K*_*D*_) to a binding isotherm (*red line*). Errors correspond to the SD of the fits. *Open symbols* correspond to control titrations into buffer. ITC, isothermal titration calorimetry; MBP, maltose-binding protein; MIRO, mitochondrial Rho GTPase; TRAK, trafficking kinesin-binding protein.

Models diverge as to whether the MIRO-TRAK interaction is regulated by Ca^2+^ binding to MIRO’s EF-hands (4) or not (2, 17, 30). This question is addressed here using quantitative ITC experiments and mutagenesis. The experiments described above were performed in the presence of 50 μM CaCl_2_. The titration of 800 μM CaCl_2_ into this protein did not reveal additional Ca^2+^ binding (Table S1), demonstrating that the EF-hands are saturated at 50 μM CaCl_2_. Lowering the CaCl_2_ concentration in the buffer 10-fold further produced the same result (Fig. 3*A*), indicating that the *K*_*D*_ for Ca^2+^ binding to both EF-hands is below 5 μM. To fully remove the Ca^2+^ from the EF-hands, MIRO1_177-591_ was dialyzed against 5 mM EGTA, 1 mM MgCl_2_, and 0.1 μM GTP. Most of the EGTA was then removed in a stepwise manner, to a final concentration of 0.08 μM, to ensure that only a small amount of the Ca^2+^ was chelated in subsequent experiments. The titration of 800 μM CaCl_2_ into this protein revealed two Ca^2+^-binding sites, with affinities differing by >10-fold (*K*_*D*_ = 0.3 μM and 3.94 μM) (Fig. 3*C* and Table S1). The intracellular concentration of free Ca^2+^ fluctuates between 0.1 μM at rest and 10 μM during activation (47, 48), suggesting that one of the Ca^2+^-binding sites of MIRO1 may be constitutively occupied by Ca^2+^ whereas the other site could play a regulatory role, binding Ca^2+^ only when its local concentration increases.

These experiments showed that in the presence of 5 mM EGTA the EF-hands of MIRO1_177-591_ are free of Ca^2+^. Under these conditions, MIRO1_177-591_ bound dimeric GCN4-TRAK1_342-431_ (*K*_*D*_ = 5.13 μM, N= 1.84) and monomeric MBP-TRAK1_342-431_ (*K*_*D*_ = 8.77 μM, N= 1.09) with similar affinities and stoichiometries as observed above in the presence of Ca^2+^ (compare Fig. 2, *B* and *C* and 3, *C* and *D*). This result provides direct evidence that the interaction of MIRO1 with TRAK1 is independent of Ca^2+^ binding to MIRO1, which is consistent with some of the previous findings (2, 17, 30).

The experiments described above were all performed in the presence of 1 mM MgCl_2_. Therefore, it must be considered whether Mg^2+^ occupies the EF-hands of MIRO1 under these conditions, as previously observed in crystal structures (10, 42). The binding of Mg^2+^ to MIRO1’s EF-hands likely has physiological importance, since the free concentration of Mg^2+^ in cells ranges from 0.5 to 1.0 mM (49). Therefore, at resting free Ca^2+^ concentrations, Mg^2+^ may saturate most EF-hands, including those of MIRO1, which has led to the proposal that the Ca^2+^-dependent activation of EF-hand proteins must take into account the displacement of Mg^2+^ (and other divalent cations) from EF-hands (50, 51). Because Mg^2+^ coordinates the nucleotide in the catalytic site of the cGTPase, MgCl_2_ cannot be removed in experiments with MIRO1_177-591_, which precipitates in its absence. Therefore, to specifically disrupt the binding of cations to the EF-hands, the bidentate cation-coordinating residues E208 and E328 were independently mutated to alanine within the first or second Ca^2+^-binding EF-hands, respectively (Fig. 3, *E* and *F*, left). These mutants bound monomeric MBP-TRAK1_342-431_ with similar affinity and stoichiometry as observed above in the presence or the absence of Ca^2+^, indicating that the MIRO1-TRAK1 interaction is independent of cation binding to the EF-hands (Fig. 3, *E* and *F*, right).

Note that the order of the titrations has been deliberately inverted among the various experiments described in this work (*i.e.*, MIRO1 and TRAK1 constructs are found sometimes in the syringe and sometimes in the cell) and each ITC experiment was repeated multiple times with similar results (Table S1). Of note, the slight endothermic heats of dissociation observed in control titrations into buffer when MIRO1_177-591_ is in the syringe (*i.e.*, Figs. 2*D* and 3*D*, open symbols) are not observed when MBP-TRAK1_342-431_ is in the syringe (Fig. 3, *E* and *F*, open symbols).

### The MIRO1-TRAK1 interaction is independent of the nucleotide state of the cGTPase

Both GTPase domains of yeast MIRO (Gem1) (52) and the nGTPase of *Drosophila* MIRO (53) have been shown to be catalytically active, but whether mammalian MIRO hydrolyzes GTP is controversial. While biochemical studies suggest that mammalian MIRO hydrolyses GTP (54, 55), key catalytic residues present in Ras-family GTPases are missing in the GTPase domains of MIRO (55). Moreover, a crystal structure of the nGTPase of MIRO1 reveals a lack of nucleotide hydrolysis in the crystals and a noncatalytic configuration of the GTP-binding site (41). Therefore, it is unclear whether the nucleotide state of MIRO1, and particularly that of the cGTPase, plays a role in target recognition. To determine the nucleotide state of MIRO1_177-591_ and its potential role in TRAK1 binding we used reverse-phase analytical HPLC and ITC.

**Figure 4:**
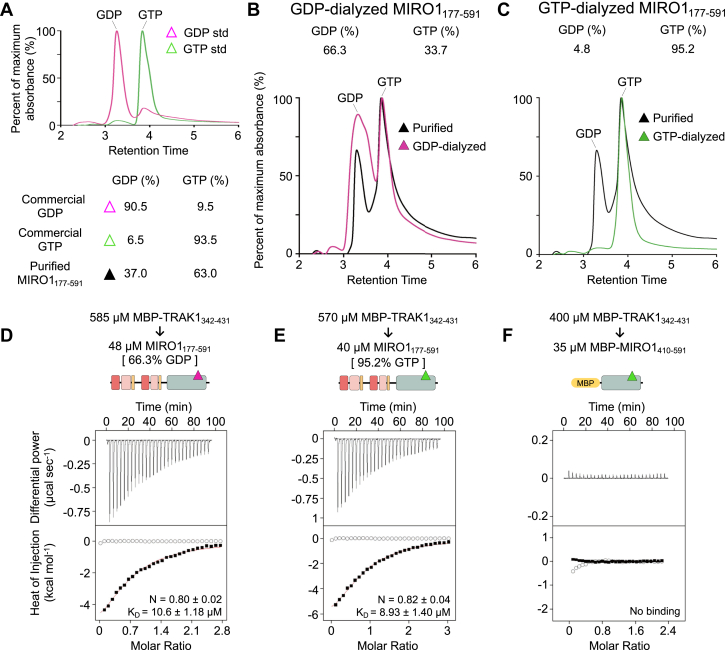
**The MIRO1-TRAK1 interaction is independent of the nucleotide state of the cGTPase**. *A*, HPLC analysis of analytical-grade commercial GDP (*pink*) and GTP (*green*) standards (*top*) and quantifications (*bottom*). The normalized maximum absorbance at 256 nm (*y*-axis) is plotted as a function of the retention time. *B* and *C*, HPLC analysis of *E*. *coli*-expressed MIRO1_177-591_ before (*black trace*) and after addition of 35-M excess of commercial nucleotide (GDP, *pink trace*; GTP, *green trace*) followed by 3-days dialysis into ITC buffer with 5 mM EGTA and no extra nucleotide added (*bottom*). Quantifications based on absorbance at 256 nm are shown above each graph and in part A (*top*). *D–F*, representative ITC titrations from N = 3 independent experiments (Table S1) of MBP-TRAK1_342-431_ into the nucleotide-exchanged MIRO1_177-591_ samples (GDP, part *B* and *D*; GTP, part *C* and *E*) or MIRO1_410-591_ (same buffer as in part *C*). Listed with each titration are the concentrations of the protein in the syringe and in the cell and the parameters of the fit (stoichiometry N and dissociation constant *K*_*D*_) to a binding isotherm (*red line*). Errors correspond to the SD of the fits. *Open symbols* correspond to control titrations into buffer. ITC, isothermal titration calorimetry; MBP, maltose-binding protein; MIRO, mitochondrial Rho GTPase; TRAK, trafficking kinesin-binding protein.

First, the nucleotide state of *E*. *coli*-expressed MIRO1_177-591_ was determined using as a reference analytical-grade commercial GDP and GTP. By reverse-phase analytical HPLC, the commercial GDP sample contains 90.5% GDP and 9.5% GTP whereas the commercial GTP sample contains 6.5% GDP and 93.5% GTP (Fig. 4*A*). Using the HPLC elution profile of these two samples as reference, *E. coli*-expressed MIRO1_177-591_ was found to contain 37.0% GDP and 63.0% GTP. To exchange the nucleotide on MIRO1_177-591_, a 35-M excess of commercial nucleotide (GDP or GTP) was added and incubated for 1 hour on ice, followed by 3-days dialysis into a buffer with no extra nucleotide added. After dialysis and passage through a PD10 column (see Experimental procedures), the sample preincubated with GDP had 66.3% GDP and 33.7% GTP (Fig. 4*B*), whereas the sample preincubated with GTP had 4.8% GDP and 95.2% GTP (Fig. 4*C*). The inefficient incorporation of GDP contrasts with the nearly complete incorporation of GTP and suggests that the cGTPase has a marked preference for GTP, analogous to other Ras-family GTPases (56). Moreover, because the cellular concentration of GTP is ∼10-fold higher than that of GDP (57), purified MIRO1_177-591_ could have been expected to be mostly GTP bound. That purified MIRO1_177-591_ had 37% GDP bound suggests that GTP was being slowly hydrolyzed during purification, as suggested by studies that show that the MIRO1 GTPases are catalytically active (54, 55). MBP-TRAK1_342-431_ bound the MIRO1_177-591_ samples preincubated with GDP or GTP with similar affinities and stoichiometries (Fig. 4, *D* and *E*), which were also similar to those observed in other experiments performed here (Figs. 2*D* and 3, *D*–*F*). From these results, we conclude that the interaction with TRAK1 is independent of the nucleotide state of the cGTPase, consistent with studies showing that mutations of the cGTPase have little effect on mitochondrial trafficking and TRAK recruitment to mitochondria (2, 35).

MIRO1_177-591_ contains both the EF hands and cGTPase. We asked whether the TRAK1-binding site was fully contained within one of these domains or whether the two domains contributed together toward the interaction. Therefore, constructs MBP-MIRO1_177-404_ (EF hands) and MBP-MIRO1_410-591_ (cGTPase) were expressed to test binding to TRAK1 by ITC. However, while MBP-MIRO1_410-591_ was well-behaved, MBP-MIRO1_177-404_ could not be used as it was poorly expressed and prone to aggregation. By ITC, MBP-MIRO1_410-591_ did not bind MBP-TRAK1_342-431_ (Fig. 4*F*). This result shows that the presence of the EF-hands is required for TRAK1 binding, but we cannot rule out participation of the cGTPase in the interaction via a combined interface with the EF-hands.

### A conserved motif in TRAK1 mediates the interaction with MIRO1 *in vitro* and in cells

**Figure 5:**
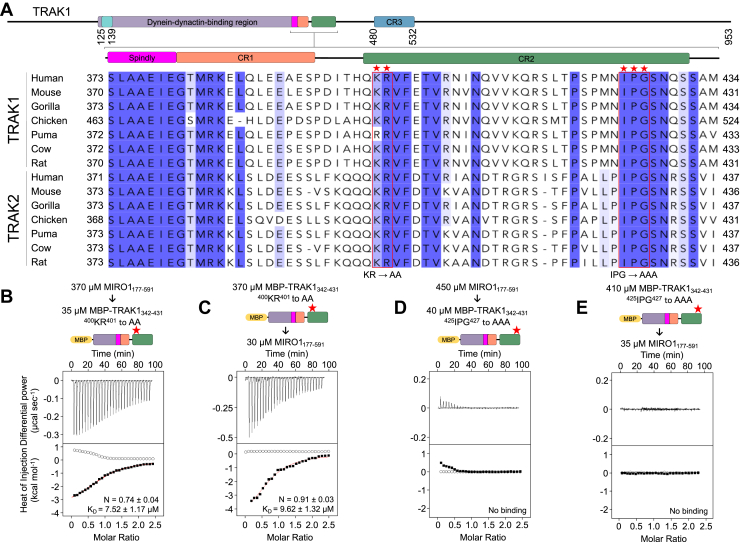
**A conserved motif in TRAK1 mediates the interaction with MIRO1**. *A*, subgroup of TRAK1/2 sequences extracted from an alignment of 183 TRAK sequences (Fig. S1) and showing residues 373 to 434, including the CR2 region that binds MIRO1 (Fig. 2, *B* and *C*). Amino acids conserved in >50% and >85% of the 183 sequences are highlighted *light* and *dark blue*, respectively. Stars highlight residues ^400^KR^401^ and ^425^IPG^427^ (*boxed red*) mutated to alanine. *B*–*E*, representative ITC titrations of MIRO1_177-591_ into the MBP-TRAK1_342-431_ mutants (*B* and *D*) and inverted titrations (*C* and *E*) from N = 3 independent experiments (Table S1). Prior to each titration, the proteins in the syringe (*top*) and in the cell (*bottom*) were codialyzed for 3 days in ITC buffer with 5 mM EGTA. Listed with each titration are the concentrations of the protein in the syringe and in the cell and the parameters of the fit (stoichiometry N and dissociation constant *K*_*D*_) to a binding isotherm (*red line*). Errors correspond to the SD of the fits. *Open symbols* correspond to control titrations into buffer. ITC, isothermal titration calorimetry; MBP, maltose-binding protein; MIRO, mitochondrial Rho GTPase; TRAK, trafficking kinesin-binding protein.

The interaction with MIRO1 was mapped above to CR2 of TRAK1 (Fig. 2). Using site directed mutagenesis, we tested the role of conserved residues within CR2 in the interaction. Among 183 TRAK sequences from different species and isoforms, two segments of high sequence conservation are observed toward the N- and C-terminal ends of CR2: ^399^QKRVFETV^406^ and ^425^IPGSN^429^ (Fig. 5*A*). Two MBP-TRAK1_342-431_ mutants, ^400^KR^401^ to AA and ^425^IPG^427^ to AAA, were made to test the role of these conserved segments in the interaction with MIRO1_177-591_. Because the interaction is independent of Ca^2+^ (Fig. 3), experiments were performed in the presence of 5 mM EGTA, which we have found favors MIRO1_177-591_ solubility. Binding of MIRO1_177-591_ to MBP-TRAK1_342-431_ was unaffected by the ^400^KR^401^ to AA mutation, independent of which protein was in the syringe and the cell (Fig. 5, *B* and *C*). In contrast, the ^425^IPG^427^ to AAA mutation eliminated binding of MIRO1_177-591_ to MBP-TRAK1_342-431_ (Fig. 5*D*), and the same result was obtained when the titration was inverted (Fig. 5*E*).

**Figure 6:**
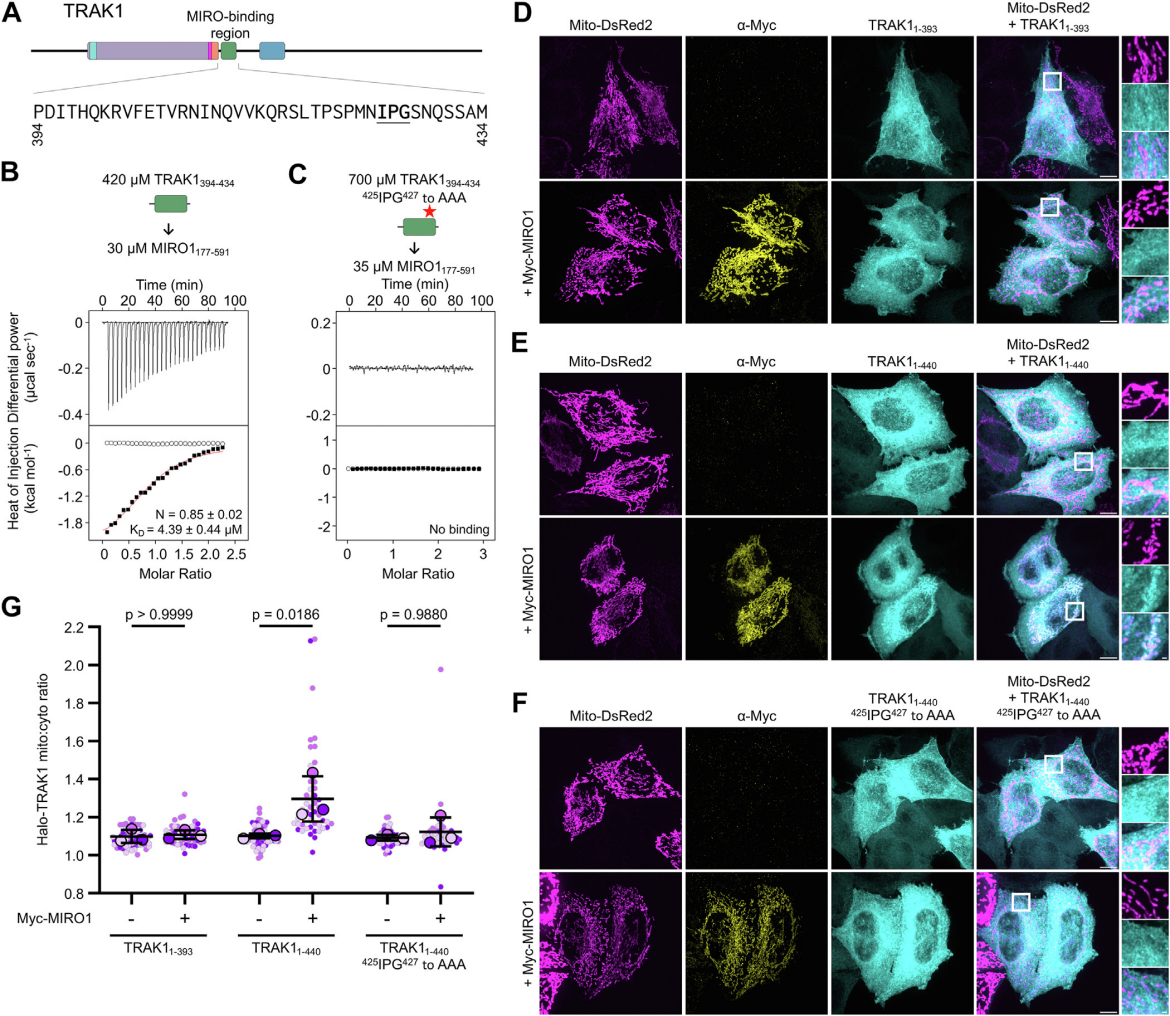
**The CR2 region of TRAK1 is sufficient to bind MIRO1 *in vitro* and in cells**. *A*, schematic and sequence representation of the MIRO1-binding region of TRAK1. *B* and *C*, representative ITC titrations of TRAK1_394-434_ into MIRO1_177-591_ WT (*B*) and mutant ^425^IPG^427^ to AAA (*C*) from N = 3 independent experiments (Table S1). Proteins were dialyzed in ITC buffer with 5 mM EGTA and 0.1 μM GTP. Proteins were dialyzed in ITC buffer with 50 μM GTP (*F*). Listed with each titration are the concentrations of the protein in the syringe and in the cell and, for interacting proteins, the parameters of the fit (stoichiometry N and dissociation constant *K*_*D*_) to a binding isotherm (*red line*). Errors correspond to the SD of the fits. *Open symbols* correspond to control titrations into buffer. *D–F*, representative maximum-intensity projections of Halo-TRAK1_1-393_ (*D*), Halo-TRAK1_1-440_ (*E*) or Halo-TRAK1_1-440_^425^IPG^427^ to AAA (*F*) and Mito-DsRed2 in HeLa cells, with or without Myc-MIRO1 coexpression. *G*, ratio of mitochondrial to cytoplasmic intensity for Halo-TRAK1 constructs, with or without Myc-MIRO1 coexpression. Data points are color-coded by experimental replicate, with smaller points representing individual cells. N = 3 independent experiments, with 48 to 63 cells per condition. The *center line* and *bars* represent the mean ± SD from independent experiments. *p*-values from one-way ANOVA with the Tukey’s multiple comparisons test are shown. The scale bars represent 10 μm (*D–F*), 1 μm (*D–F* insets). ITC, isothermal titration calorimetry; MIRO, mitochondrial Rho GTPase; TRAK, trafficking kinesin-binding protein.

Based on these results, we made a shorter TRAK1 peptide narrowly encompassing CR2 (TRAK1_394-434_) (Fig. 6*A*). TRAK1_394-434_ bound MIRO1_177-591_ with stoichiometry and affinity consistent with other experiments described here (Fig. 6*B*), and the interaction was abolished when the ^425^IPG^427^ to AAA mutation was introduced into this peptide (Fig. 6*C*).

Next, we assessed the physiological importance of this interaction for TRAK1 recruitment to mitochondria in cells. Two N terminally Halo-tagged constructs, one lacking (TRAK1_1-393_) and one containing (TRAK1_1-440_) the CR2 region necessary for MIRO1 binding *in vitro*, were individually expressed in HeLa cells and visualized using confocal microscopy. Mitochondria were labeled with Mito-DSRed2, and the overlap of each TRAK1 construct with mitochondria was quantified in the presence or the absence of Myc-MIRO1, whose overexpression is known to be required for efficient mitochondrial recruitment of exogenously expressed TRAK1 (34). TRAK1_1-440_ but not TRAK1_1-393_ colocalized with mitochondria and only with Myc-MIRO1 coexpression (Fig. 6, *D*, *E*, and *G*). Moreover, TRAK1_1-440_ recruitment to mitochondria was abolished by the ^425^IPG^427^ to AAA mutation (Fig. 6, *F* and *G*). These results suggest that the CR2 of TRAK1 is necessary for MIRO1-dependent recruitment of TRAK1 to mitochondria.

## Discussion

**Figure 7:**
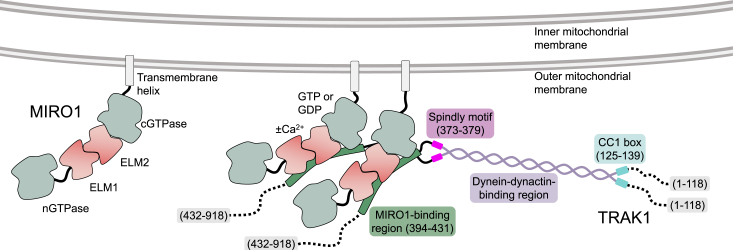
**Two MIRO1 molecules bind one TRAK1 dimer on the mitochondrial surface.** One TRAK1 dimer binds to two MIRO1 molecules on the mitochondrial surface, independent of Ca^2+^-binding to the EF-hands or the cGTPase nucleotide state of MIRO1. The interaction is mediated by a conserved motif C terminal to the Spindly motif of TRAK1 and directly implicates MIRO1’s EF-hands. MIRO, mitochondrial Rho GTPase; TRAK, trafficking kinesin-binding protein.

MIRO plays a central role in mitochondrial trafficking by recruiting TRAK, which in turn coordinates anterograde and retrograde motility along microtubules by kinesin-1 and dynein-dynactin (8). It is, however, poorly understood and often debated how mitochondrial trafficking is regulated and specifically whether the MIRO-TRAK interaction is responsible for this regulation. Here, we have mapped the MIRO1-TRAK1 interaction and analyzed the effect of MIRO1-binding cofactors (Ca^2+^, Mg^2+^, GTP, and GDP) on TRAK1 binding. While this work focused on isoforms MIRO1 and TRAK1, the findings can likely be extended to MIRO2 and TRAK2, since sequence conservation was used as a guiding principle in mapping the interaction of the two proteins. The main findings of this study are the following: (a) MIRO1 binds to a conserved motif on TRAK1, located C terminal to the Spindly motif and consisting of TRAK1 residues 394 to 431, (b) mutations in this sequence disrupt MIRO1 binding *in vitro* and mitochondrial localization of TRAK1 in cells, (c) TRAK1 binds to a fragment containing MIRO1’s EF-hands and cGTPase, (d) deleting MIRO1’s EF-hands abolishes the interaction, pointing to their direct involvement in TRAK1 binding either alone or together with the cGTPase, (e) the MIRO1-TRAK1 interaction is independent of the nucleotide state of the cGTPase, (f) the interaction is also independent of Ca^2+^ or Mg^2+^ binding to MIRO1’s EF-hands, (g) the interaction has 1:1 stoichiometry, such that one TRAK1 dimer has the potential to bind two MIRO1 molecules on the mitochondrial surface (Fig. 7). Therefore, this interaction may be further enhanced through avidity via the formation of MIRO clusters on the mitochondrial membrane (7). These findings are consistent with studies that have found that the MIRO-TRAK interaction is independent of Ca^2+^ binding to MIRO’s EF-hands (17, 30, 39).

Local increases in Ca^2+^ concentration stop mitochondrial trafficking (36, 37, 38), and this effect is suppressed by mutations in MIRO’s EF-hands (4, 30, 39, 40). Because TRAK acts as an intermediate scaffold between MIRO and the microtubule-based motors that drive mitochondrial trafficking (2, 14), it is reasonable to think that the effect of Ca^2+^ on mitochondrial motility could be through regulation of the MIRO-TRAK interaction (4). However, our finding that the MIRO1-TRAK1 interaction is independent of Ca^2+^ binding to MIRO1 suggests that a different mechanism is responsible for the Ca^2+^-dependent arrest of mitochondria. For instance, some evidence has suggested that kinesin-1 binds MIRO1 directly and that this interaction is regulated by Ca^2+^ (30, 39). The binding of kinesin-1 presumably implicates the EF-hands of MIRO1 and is thought to be independent of the MIRO1-TRAK1 interaction demonstrated here. While our data cannot rule out this possibility, we note that this model cannot explain why retrograde motility by dynein-dynactin is also arrested by increases in Ca^2+^. Moreover, recent results negate the existence of a direct MIRO1-kinesin-1 interaction (17). Other models propose that Ca^2+^ regulates MIRO’s interactions with other proteins, which in turn control mitochondrial trafficking and dynamics, such as syntaphilin (17), Armcx (58, 59), and MFN (60). This may explain why mutations in MIRO’s EF-hands impair Ca^2+^ regulation.

Our work implicates the EF-hands of MIRO1 in TRAK1 binding. Most EF-hand proteins undergo major conformational changes upon Ca^2+^ binding, with Ca^2+^ acting as an on/off switch for target recognition (61). Thus, it may appear surprising that Ca^2+^ (or Mg^2+^) binding to MIRO1’s EF-hands has little effect on the interaction with TRAK1. Indeed, our data suggests that MIRO1 acts as a Ca^2+^ sensor, but probably not through a major conformational change. Under resting conditions, when the intracellular free Ca^2+^ concentration is ∼0.1 μM, one of the EF-hands of MIRO1 is occupied by Ca^2+^ whereas the other EF-hand may bind Ca^2+^ only upon activation, when the free Ca^2+^ concentration can reach ∼10 μM (47, 48). In other words, the two pairs of EF-hands of MIRO1 can be separated into constitutively-bound and regulatory Ca^2+^-binding pairs. At rest, the regulatory pair (which our data does not assign) is likely occupied by Mg^2+^, whose free intercellular concentration ranges from 0.5 to 1.0 mM (49), such that Ca^2+^ binding may proceed through the displacement of Mg^2+^, as proposed for other EF-hand proteins (50, 51). While MIRO1 may share some of these features with other EF-hand-containing proteins, other characteristics make it unique. For instance, MIRO’s EF-hands differ substantially from those of the prototypical Ca^2+^-sensing protein calmodulin (62). In calmodulin, all four EF-hands bind Ca^2+^, which induces a conformational change that exposes hydrophobic pockets in the two pairs of EF-hands for the binding of calmodulin’s cellular targets (62). In MIRO1, only one EF-hand of each pair binds Ca^2+^. Moreover, in calmodulin, the two pairs of EF-hands are separated by a long and flexible linker, allowing them to move with relative independence of one another to grab the target protein (62). In MIRO, however, the two pairs of EF-hands are rigidly packed against one another (10, 42). Finally, unlike calmodulin, MIRO already has target peptides bound intramolecularly to the hydrophobic pockets of the two pairs of EF-hands, *i.e.* the ligand-mimic helices (10) (Figs. 1*A* and 3, *E* and *F*). These differences suggest that, while in calmodulin the EF-hands are used as a Ca^2+^-dependent switch for target recognition, in MIRO the EF-hands play a structural role, acting as a rigid domain. This may explain why MIRO1’s EF-hands show fundamentally the same conformation in crystal structures determined under different conditions (apo, Ca^2+^- or Mg^2+^-bound) (10, 42). In summary, MIRO seems to function as a Ca^2+^-dependent sensor, but not a Ca^2+^-dependent conformational switch.

## Experimental procedures

### Proteins

Primers used in this study are listed in Table S2. The complementary DNA coding for human MIRO1 (UniProt ID: Q81X12) was purchased from Addgene (Plasmid: 127613). The complementary DNA coding for human TRAK1 (Uniprot ID: Q9UPV9) and GCN4-TRAK1_342-431_ were synthesized with codon optimization for *E. coli* expression by GenScript (Piscataway, NJ). MIRO1_1-591_ and TRAK1_99-532_ were cloned into vector pRSFDuet-1 and MIRO1_177-591_, GCN4-TRAK1_342-393_, and GCN4-TRAK1_342-431_ were cloned into vector pETDuet-1 (Novagen). MBP-MIRO1_1-180_, MBP-MIRO1_410-591_, MBP-TRAK1_342-431_, and TRAK1_394-434_ (WT and ^425^IPG^427^ to AAA) and were cloned into vector pMAL-c6T (New England Biolabs). Point mutations in MIRO1_177-591_ (E208A and E328A) and MBP-TRAK1_342-431_ (^400^KR^401^ to AA and ^425^IPG^427^ to AAA) were introduced using the QuikChange mutagenesis kit (Agilent Technologies). The Myc-MIRO1 construct was obtained from Addgene (Plasmid: 47888). Halo-TRAK1 1 to 393 and Halo-TRAK1 1 to 440 (WT and mutant ^425^IPG^427^ to AAA) were cloned from HA-TRAK1 (a gift from C. Hoogenraad, Utrecht University) into pFN21A-HaloTag-CMV vector (Promega). Affinity tags used in purification (see below) are either part of the vectors or were added during cloning.

All the proteins were expressed in *E. coli* ArcticExpress(DE3) cells (Agilent Technologies), grown in terrific broth medium at 37 °C to an *A*_600_ of 1.5 to 2, followed by 24 h at 9 °C with the addition of 0.35 mM IPTG. Cells were pelleted by centrifugation, resuspended in TRAK1 or MIRO1 buffers (see below) supplemented with 1 mM PMSF and lysed using a Microfluidizer (Microfluidics). Lysates were clarified by centrifugation and supernatants were loaded onto their corresponding affinity columns according to protocols from the manufactures (see below).

All the TRAK1-derived proteins were prepared in TRAK1 buffer (20 mM Hepes pH 7.5 and 200 mM NaCl). TRAK1_99-532_ and GCN4-TRAK1 constructs were affinity-purified on a Strep-Tactin Sepharose column (IBA Lifesciences) and eluted with 40 mM biotin pH 8.0 (dissolved in TRAK1 buffer), followed by purification on a Ni-NTA column (G-Biosciences). MBP-TRAK1_342-431_ constructs (WT and mutants) were affinity-purified on an amylose column (New England Biolabs), and further purified through a Strep-Tactin column. MBP-TRAK1_394-434_ peptides (WT and mutant) were purified through an amylose affinity column (New England Biolabs). The MBP tag was removed by incubation with Tobacco Etch Virus protease for 48 h at 4 °C. The cleaved peptides were separated from 6xHis-MBP and 6xHis-Tobacco Etch Virus on a Ni-NTA column. After tag removal, the peptides were further purified on an SD75HL 16/60 column (GE HealthCare) in 20 mM Hepes pH 7.5 and 50 mM NaCl. The peptides were loaded onto a SourceQ anion exchange column (Cytiva) and eluted in the flowthrough in 20 mM Hepes pH 7.5.

All the MIRO1-derived proteins were prepared in MIRO1 buffer (20 mM Hepes pH 7.5, 300 mM NaCl, 1 mM CaCl_2_, 1 mM MgCl_2_, 5% w/v sucrose, and 0.1 μM GTP). MIRO1_1-591_ was prepared in MIRO1 buffer containing 500 mM NaCl, 30 mM imidazole pH 7.3, and 5% glycerol. The protein was affinity-purified on a Ni-NTA column and then on a Strep-Tactin Sepharose column and eluted with 40 mM Biotin pH 8.0 (dissolved in the same buffer). MBP-MIRO1_1-180_ (in MIRO1 buffer lacking CaCl_2_) was purified through a Strep-Tactin column and eluted with 40 mM biotin pH 8.0 and diluted to a NaCl concentration of 50 mM. The protein was further purified on a MonoQ anion exchange column (Cytiva) with Buffer A (20 mM Hepes pH 7.5, 50 mM NaCl, 1 mM MgCl_2_, and 5% w/v sucrose) and eluted using a stepwise gradient of Buffer B (Buffer A + 1 M NaCl). MIRO1_177-591_ (WT and mutants) were purified on a Ni-NTA column in MIRO1 buffer supplemented with 30 mM imidazole pH 7.3. Aggregates were removed by centrifugation at 372,000*g* for 20 min. The protein was diluted to a NaCl concentration of 75 mM, loaded onto a SourceQ column, and eluted in the flowthrough in MIRO1 buffer lacking NaCl (contaminants remain bound to the column under these conditions). MBP-MIRO1_410-591_ (in MIRO1 buffer lacking CaCl_2_) was affinity purified on an amylose column (New England Biolabs).

### Glycerol gradient cosedimentation

Proteins were mixed at a final concentration of 4 μM in starting buffer (20 mM Hepes pH 7.5, 500 mM NaCl, 1 mM MgCl_2_, 1 mM CaCl_2_, 1 mM DTT, and 0.1 μM GTP). A 5%–30% glycerol gradient was prepared by layering 2 ml of light solution (starting buffer + 5% glycerol) on top of 2 ml of heavy solution (starting buffer + 30% glycerol) and mixing the solutions using a BioComp Gradient Master 107 (Biocomp Instruments) set to rotate for 94 s at 86 degrees. Proteins (250 μl) were pipetted on top of the gradient and spun using a SW-60 rotor at 165,000*g* for 16 h at 4 °C. Samples were fractionated and analyzed by SDS-PAGE. Gels were Coomassie blue-stained, captured with the program GENESys V1.5.6.0 (Genesys Limited) set to capture a visible protein gel stained with Coomassie Blue, and band intensities were quantified using ImageLab (Bio-Rad).

### Isothermal titration calorimetry

ITC measurements were carried out on a VP-ITC instrument (MicroCal). Protein samples were dialyzed against ITC buffer (20 mM Hepes pH 7.5, 400 mM NaCl, 1 mM MgCl_2_, 5% w/v sucrose, 0.125 mM tris(2-chloroethyl) phosphate, and 0.1 μM GTP) with the addition of experiment-specific cofactors (CaCl_2_, GDP, and EGTA) for at least 3 days. Protein concentrations were determined using the Bradford method. Peptide concentrations were measured using fluorescence after labeling with fluorescamine. Proteins in the syringe were titrated at a concentration 8- to 15-fold higher than that of proteins in the cell of volume 1.44 ml. Experiments were carried out at 25 °C. Titrations consisted of 10 μl injections, lasting for 10 s, with an interval of 200 s between injections. Heats of binding were determined from the point-by-point subtraction of the heats of injection into buffer (control) from the heats of injection into protein. Experiments were repeated (see Table S1). The program Origin (OriginLab) was used to analyze the data and fit binding curves. The experiment-specific cofactors and parameters of the fits are listed in the figures and Table S1.

### Analysis of bound nucleotide

Proteins were loaded onto a Sephadex G-25 PD10 column (Global Life Science Solutions) with 20 mM Tris pH 7.5 to remove unbound nucleotide, and fractions (500 μl each) were collected. Peak fractions were denatured with the addition of 2.5 μl of 10% perchloric acid followed by 2.5 μl of 4 M sodium acetate pH 4.0. Denatured proteins were pelleted by centrifugation. The supernatants (200 μl), containing the nucleotides, were mixed with 100 μl of HPLC loading buffer (100 mM KH2PO4, 100 mM K2HPO4, and 10 mM tetrabutylammonium bromide pH 6.5), and 250 μl was run isocratically through a Vydac 208MS C8 reverse phase HPLC column (VWR) with loading buffer supplemented with 8.5% acetonitrile. Similarly, commercial nucleotides were solubilized in 20 mM Tris pH 7.5 to a final concentration of 10 μM. A 200 μl volume was mixed with 100 μl of HPLC loading buffer, and 250 μl were run isocratically through a Vydac 208MS C8 reverse phase HPLC column (VWR) with loading buffer supplemented with 8.5% acetonitrile. To exchange the nucleotide on MIRO1_177-591_, a 35-M excess of commercial nucleotide (GDP or GTP) was added and incubated for 1 hour on ice, followed by a 3-day dialysis into a buffer with no extra nucleotide added.

### Mass photometry

Mass photometry experiments were performed on a Refeyn TwoMP-0220 instrument using programs AcquireMP v2022 R1 and DiscoverMP v2022 R1 for analysis. Proteins were dialyzed for 24 h against 20 mM Hepes pH 7.5, 300 mM NaCl, 1 mM CaCl_2_, and 1 mM MgCl_2_. Two hours before the experiments, proteins were diluted to a concentration of 600 nM (*i.e.*, a >12-fold dilution) in a buffer containing 100 mM NaCl, 50 μM MgCl_2_, 50 μM GTP and either 1 mM EGTA or 100 μM CaCl_2_. The instrument was calibrated with the corresponding buffer and proteins were applied to a final concentration of 125 to 150 nM. Movies were acquired for 60 s using default parameters. Ratiometric contrasts were converted to molecular weights using a mass calibration curve generated from three proteins: 75 nM bovine serum albumin (MW: 66.43 kDa), 75 nM beta-amylase (MW: 112 kDa dimer and 224 kDa tetramer), and 22.5 nM thyroglobulin (MW: 670 kDa). Gaussian curves were fit to each histogram distribution, and the masses and normalized counts were determined. For each fit, the bin width was set to 1 kDa, and the data are reported as the percent relative to the bin with the highest number of counts.

### Sequence analysis

Vertebrate TRAK and MIRO sequences were aligned with the program Clustal Omega (63) and sequence conservation scores were calculated with the program Scorecons using the valdar01 method (64) (Figs. S1, *A* and *B* and 2*A*). Coiled-coil predictions were performed with the program prabi (65) (Figs. S1*A* and 2*A*). The program Waggawagga was used for prediction of coiled-coil heptads (66).

### TRAK localization to mitochondria

HeLa-M cells (A. Peden, Cambridge Institute for Medical Research) were maintained in Dulbecco's modified Eagle's medium (Corning, 10–017-CM) supplemented with 1% GlutaMAX (Thermo Fisher Scientific, 35050061) and 10% fetal bovine serum (HyClone, SH30071.03). Cells were maintained at 37 °C in a 5% CO_2_ incubator. Cells tested negative for *mycoplasma* contamination and were authenticated by short tandem repeats profiling at the DNA Sequencing Facility of the University of Pennsylvania. For imaging experiments, cells were plated on 35 mm glass-bottom dishes (MatTek, P35G-1.5–20-C) and transfected using FuGENE 6 Transection Reagent (Promega, E269A).

Twenty-four hours after transfection, HeLa cells were fixed for 10 min using warm PBS with 4% paraformaldehyde and 4% sucrose and washed three times with PBS. Cells were labeled with 100 nM Janelia Fluor 646-Halo ligand (Promega, GA1121) in PBS for 20 min at 37 °C, followed by 30-min wash with PBS at 37 °C. Cells were then permeabilized with 0.2% Triton X-100 in PBS for 15 min, and washed three times with PBS. Cells were blocked for 1 h in blocking solution (5% goat serum and 1% bovine serum albumin in PBS) and incubated overnight at 4 °C with α-Myc antibody (Invitrogen, R950–25) diluted 1:1000 in blocking solution. Cells were then washed three times with PBS, incubated for 1 h with Alexa Fluor 488 goat anti-mouse IgG (H + L) (Invitrogen, A11029) diluted 1:1000 in blocking solution, and washed three more times with PBS.

HeLa cells were imaged using a V3 spinning disk confocal on a Nikon Eclipse Ti Microscope with an Apochromat 100 × 1.49 numerical aperture oil immersion objective (Nikon). Images were acquired with a Hamamatsu CMOS ORCA-Fusion (C11440–20UP) driven by VisiView software (Visitron, https://www.visitron.de/products/visiviewr-software.html). Z-stacks spanning the height of transfected cells were collected at 200-nm step-size.

Maximum-intensity projections were generated for the Halo-TRAK1 and Mito-DsRed2 channels in ImageJ (NIH, https://imagej.net/ij/download.html). The area of the Mito-DsRed2 channel was then converted to a binary mask using the Pixel classification module of Ilastik, a machine-learning based image segmentation program (67). The binarized image was used to make two regions of interest for each cell, one encompassing the mitochondria and one encompassing everything except the mitochondria. The mean fluorescence intensity of Halo-TRAK1 was measured in each region to determine the ratio of mitochondrial to cytoplasmic signal.

## Data availability

All the data described in this work are shown in the main text and Supporting Information.

## Supporting information

This article contains supporting information.

## Conflict of interest

The authors declare that they have no conflicts of interest with the contents of this article.
