# Supplementary material for: Interaction between the mitochondrial adaptor MIRO and the motor adaptor TRAK
Source: J Biol Chem. 2023 Nov 8;299(12):105441. doi: 10.1016/j.jbc.2023.105441 (PMC10746525; doi:10.1016/j.jbc.2023.105441)
Supplement: Supporting information [file mmc1.pdf]

Supporting Information for

Interaction between the mitochondrial adaptor MIRO and the motor adaptor TRAK

Authors: Elana E. Baltrusaitis[1,2,‡], Erika E. Ravitch[1,‡], Adam R. Fenton[1,3], Tania A. Perez[1,3], Erika L. F. Holzbaur[1,2,3], and Roberto Dominguez[1,2,*]

Affiliations

[1] Department of Physiology, Perelman School of Medicine, University of Pennsylvania, Philadelphia, Pennsylvania, USA.

[2] Biochemistry and Molecular Biophysics Graduate Group, Perelman School of Medicine, University of Pennsylvania, Philadelphia, Pennsylvania, USA.

[3] Cell and Molecular Biology Graduate Group, Perelman School of Medicine, University of Pennsylvania, Philadelphia, USA.

‡ These authors contributed equally to this work.

* Corresponding author: droberto@pennmedicine.upenn.edu

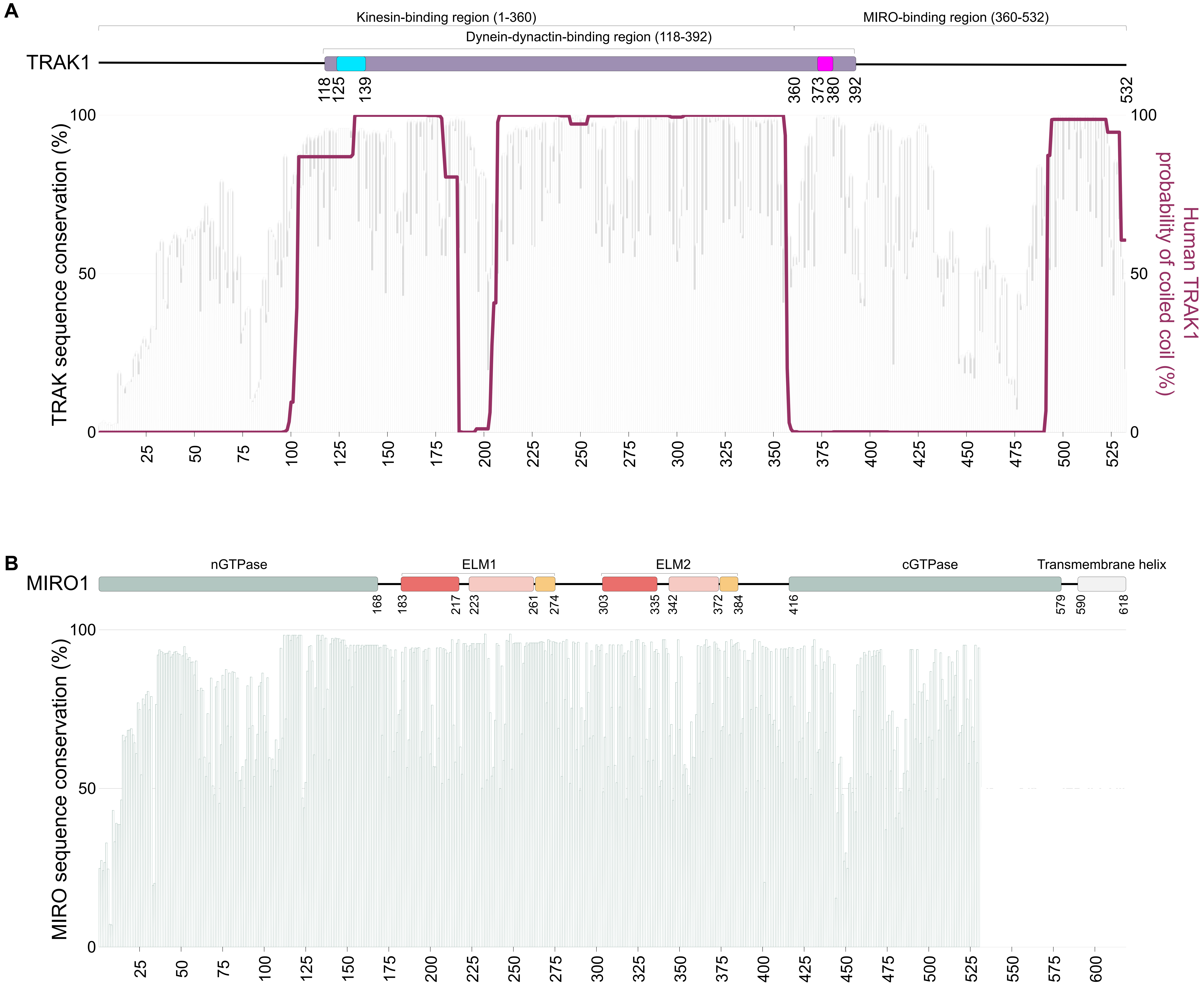

Figure S1. Sequence conservation and coiled coil-prediction. *A,* Per-amino acid sequence conservation and coiled-coil prediction scores for the 1-532 amino acid region of TRAK. Numbering is according to human TRAK1. Insertions or deletions in other sequences relative to human TRAK1 are not shown. A diagram on top shows domains and interactions within this region of TRAK (see also Fig. 1A). Sequence conservation scores (calculated with the program Scorecons, see Methods) are based on an alignment of 183 vertebrate TRAK sequences (93 TRAK1 and 90 TRAK2). The coiled-coil prediction (calculated with the program prabi, see Methods) used a 28-amino acid window. *B,* Per-amino acid sequence conservation of MIRO. Numbering is according to human MIRO1. Insertions or deletions in other sequences relative to human MIRO1 are not shown. The diagram on top shows MIRO1's domains (see also Fig. 1A). The sequence conservation scores are based on an alignment of 188 vertebrate MIRO sequences (94 MIRO1 and 94 MIRO2).

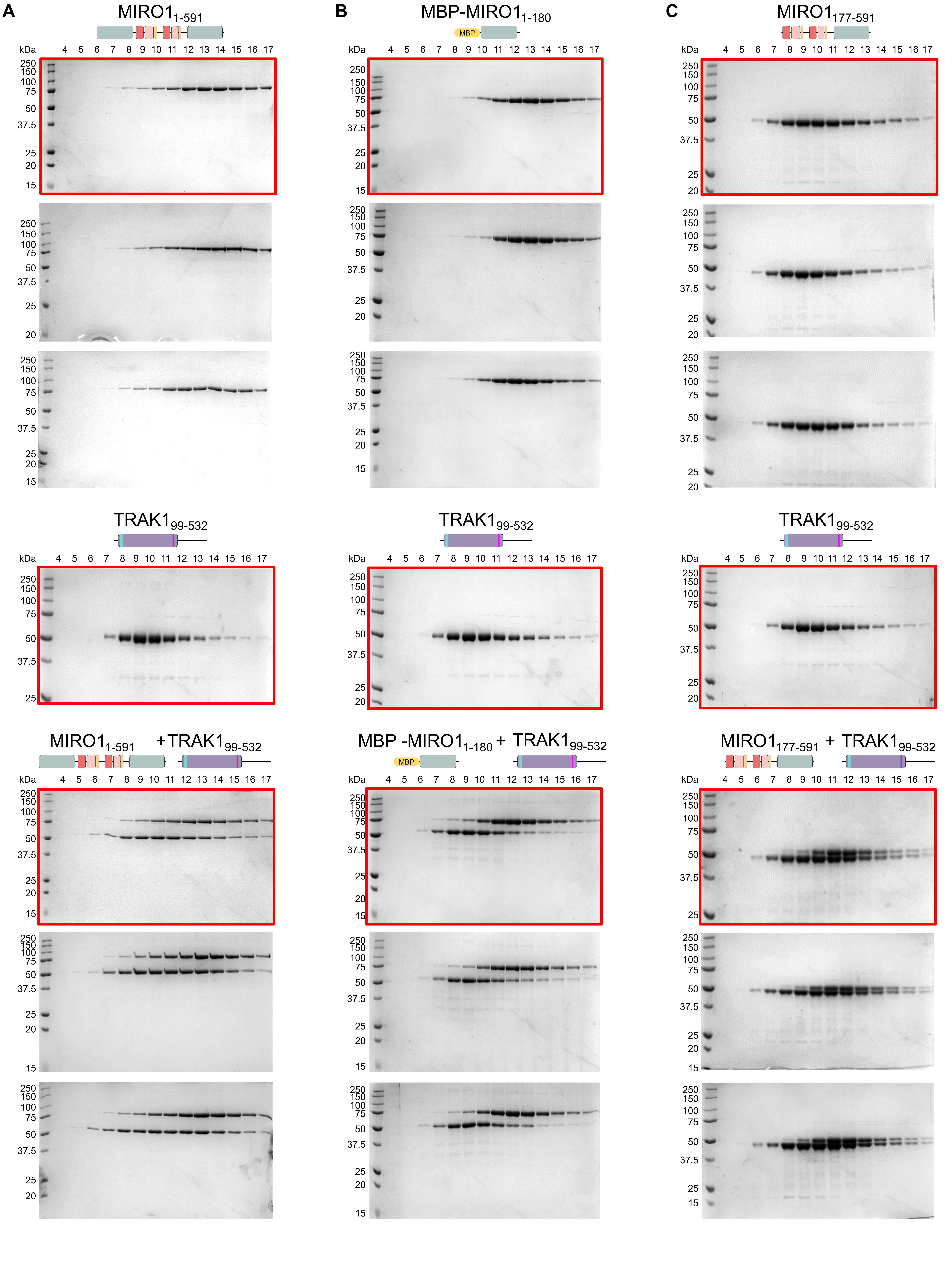

Figure S2. Interaction of TRAK1 and MIRO1 by glycerol gradient cosedimentation. SDS-PAGE analysis of glycerol gradient cosedimentation experiments of MIRO1 constructs alone, TRAK1 constructs alone, and TRAK1 with MIRO1 constructs (as indicated). Glycerol gradient fractions are indicated above each lane. Gels framed in red are also shown in Fig. 1B-D, and all gels shown here were used in statistical analyses shown in Fig. 1B–D.

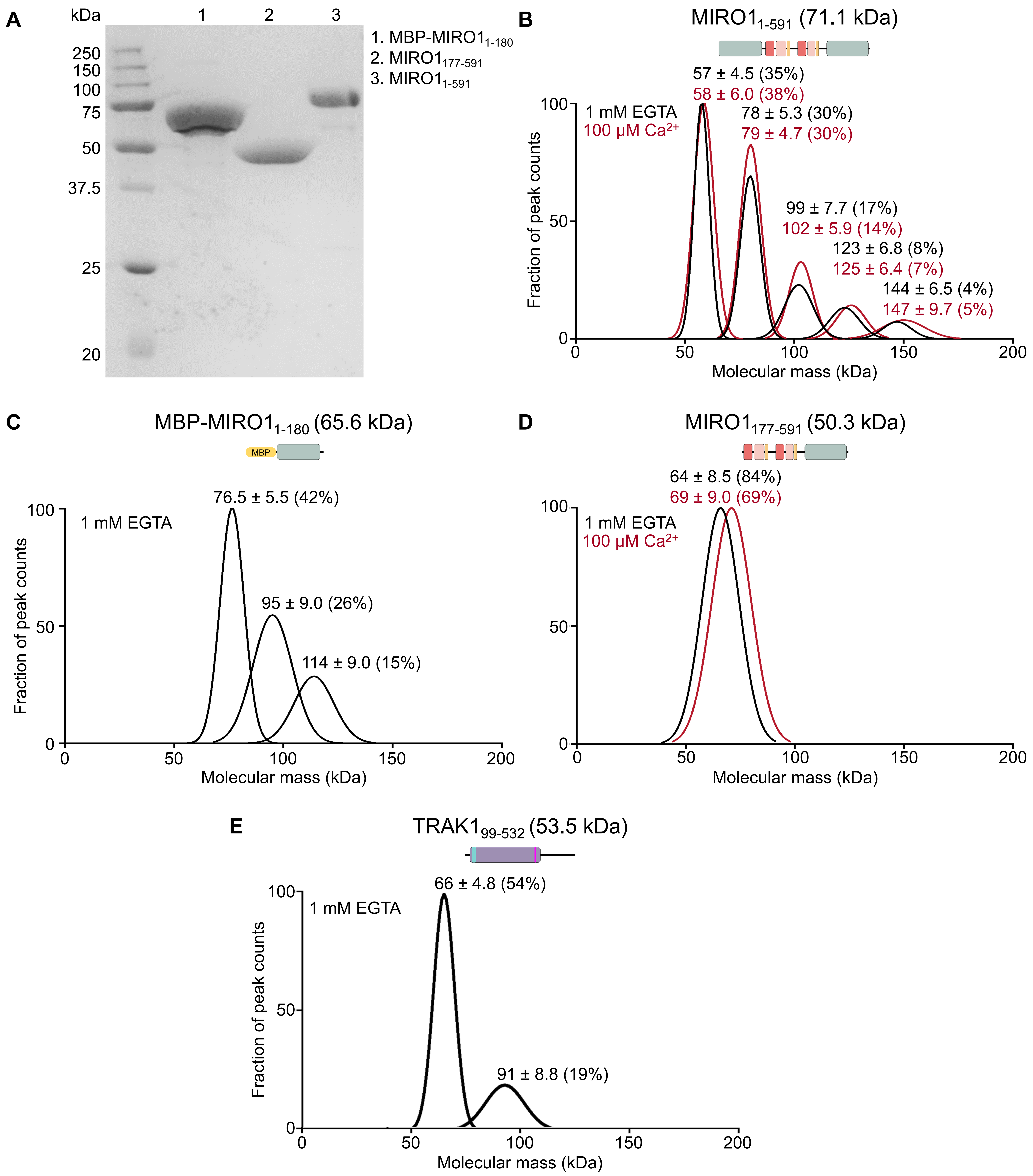

Figure S3. Mass photometry analysis of MIRO1 and TRAK1 constructs. *A*, 12% SDS-PAGE of MIRO1 constructs MIRO1$_{1\text{-}591}$, MBP-MIRO1$_{1\text{-}180}$, and MIRO1$_{177\text{-}591}$ (as indicated). *B-E*, Mass photometry analysis of the MIRO1 constructs and TRAK1$_{99\text{-}532}$ (theoretical masses shown in parentheses). MIRO1$_{1\text{-}591}$ and MIRO1$_{177\text{-}591}$, which include the EF-hands, were analyzed in the presence or the absence of Ca^{2+}. Buffer conditions for the experiments with Ca^{2+} (red curve) are: 20 mM HEPES, 100 mM NaCl, 100 μM $CaCl_2$, 50 μM $MgCl_2$, 50 μM GTP. Buffer conditions for experiments without Ca^{2+} (black curve) are: 20 mM HEPES, 100 mM NaCl, 1 mM EGTA, 50 μM $MgCl_2$, 50 μM GTP. The measured masses were binned (bin width = 1 kDa), and the graphs show the Gaussian fits of the distribution of masses normalized to the bin with the highest number of counts for each experiment. Masses were determined by ratiometric contrast, with calibrations to bovine serum albumin, β-amylase, and thyroglobulin standards (see Methods).

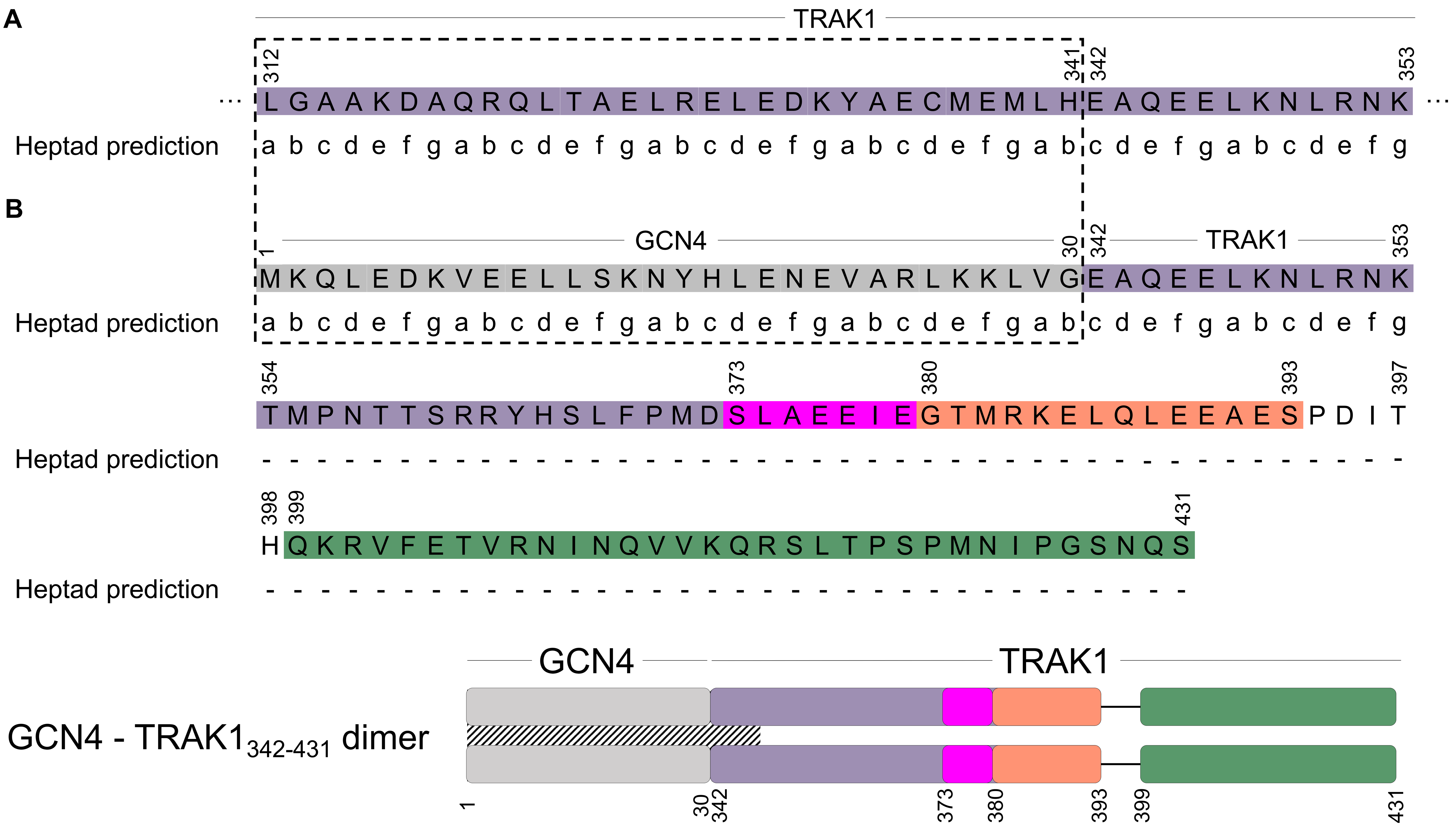

Figure S4. Design of GCN4-TRAK1 constructs. *A*, Coiled-coil heptad prediction (calculated with the program Waggawagga (see Methods)) for the 312-353 region of human TRAK1. *B*, TRAK1 amino acids in the dashed box were replaced with a 30-amino acid GCN4 leucine zipper (gray), ensuring the heptad prediction was in register for both proteins at the point of linkage. A domain diagram of the resulting GCN4-TRAK1$_{342\text{-}431}$ dimeric construct is shown at the bottom (domains colored as in Fig. 2A,B). Diagonal lines indicate the predicted region of coiled coil, which encompasses GCN4 and a short portion of TRAK1 (342-353). A shorter GCN4-TRAK1$_{342\text{-}393}$ construct was also obtained (see Fig. 2C).

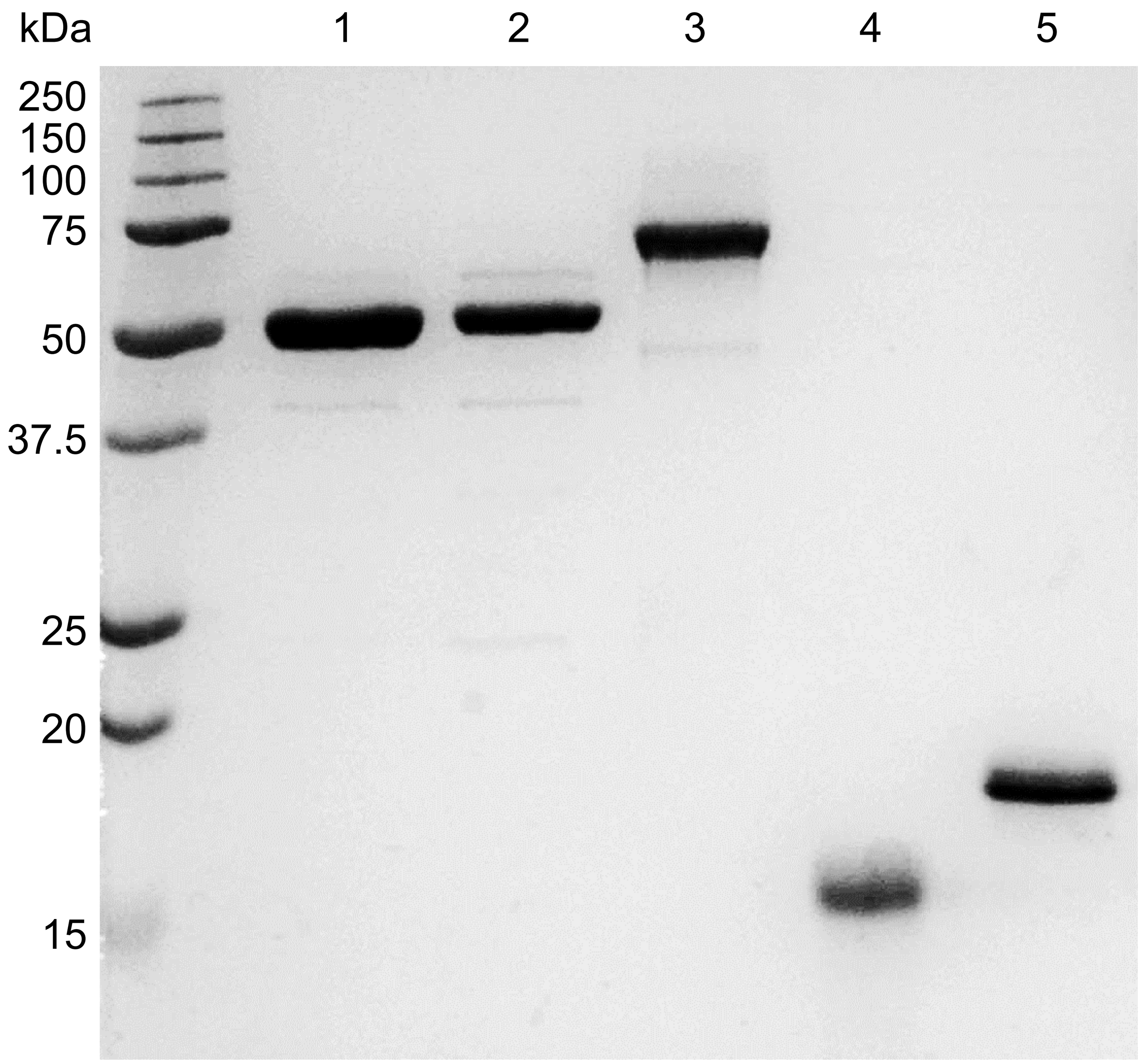

1. MIRO1$_{177\text{-}591}$ EF1 mutant (E208A) 50.3 kDa
2. MIRO1$_{177\text{-}591}$ EF2 mutant (E328A) 50.3 kDa
3. MBP-MIRO1$_{410\text{-}591}$ 65.9 kDa
4. GCN4-TRAK1$_{342\text{-}393}$ (monomer: 13.8 kDa)
5. GCN4-TRAK1$_{342\text{-}431}$ (monomer: 18.1 kDa)
6. MBP-TRAK1$_{342\text{-}431}$ (55.1 kDa)
7. MBP-TRAK1$_{342\text{-}431}$ ^{400}KR^{401} → AA (55.0 kDa)
8. MBP-TRAK1$_{342\text{-}431}$ ^{425}IPG^{427} → AAA (55.1 kDa)
9. TRAK1$_{394\text{-}434}$ (4.8 kDa)
10. TRAK1$_{394\text{-}434}$ mutant 2 (4.7 kDa)

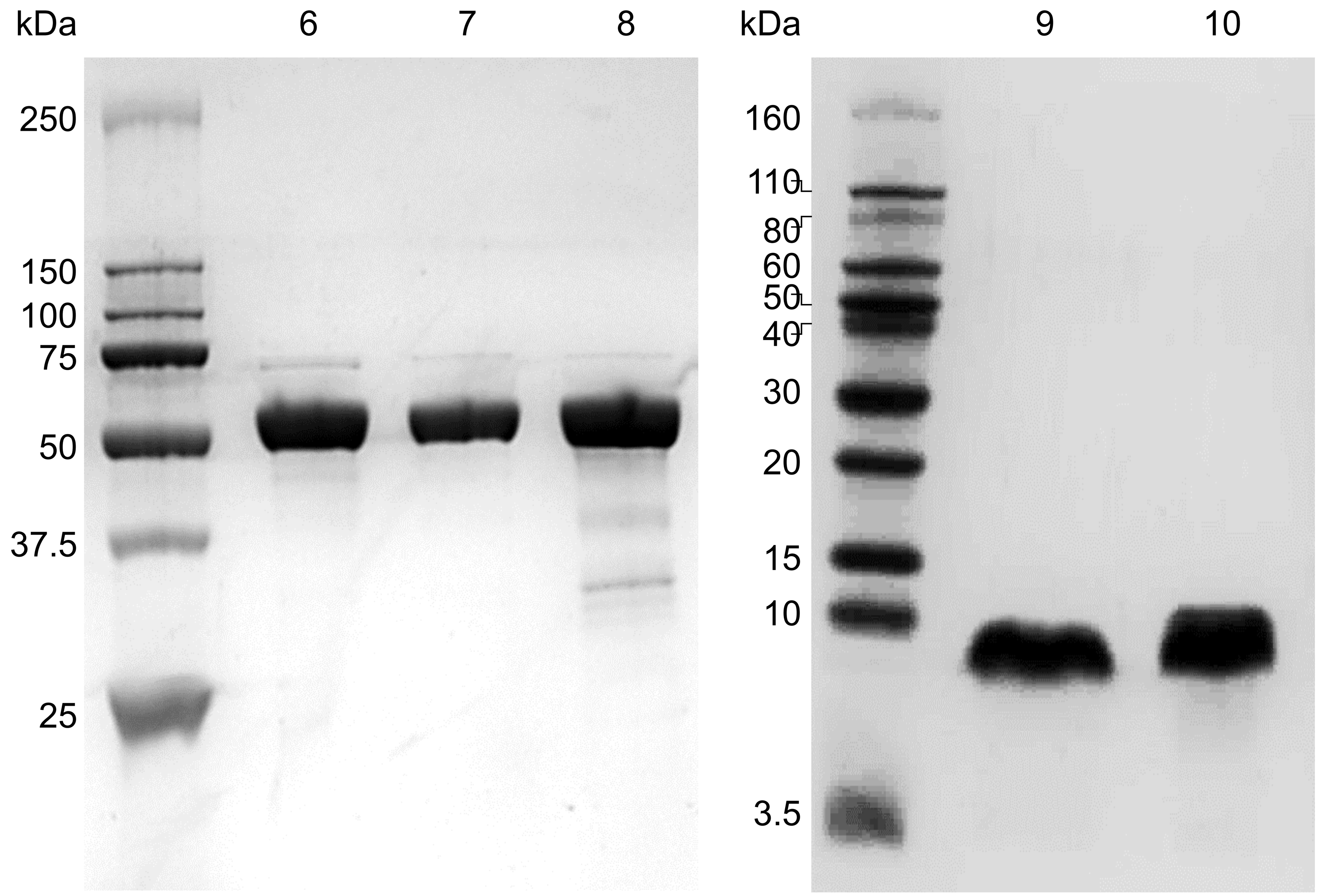

Figure S5. SDS-PAGE analysis of proteins used in this study*.* SDS-PAGE (15%) analysis of MIRO1 and TRAK1 constructs (numbered as indicated, MW in parentheses).

Table S1. ITC experiments, experimental conditions, and fitting parameters

Buffer	ITC titration (syringe → cell)	Experiment	Conc. syringe (µM)	Conc. cell (µM)	*N*	K_d (µM)	Representative experiment
50 µM $CaCl_2$, 1 mM $MgCl_2$, 0.1 µM GTP	MIRO1$_{177-591}$ → GCN4-TRAK1$_{342-431}$	1	300	15	1.56 ± 0.07	9.35 ± 1.29	Fig. 2B
		2	310	16	1.93 ± 0.02	7.04 ± 0.43	
		3	300	15	1.59 ± 0.05	9.35 ± 0.96	
50 µM $CaCl_2$, 1 mM $MgCl_2$, 0.1 µM GTP	MIRO1$_{177-591}$ → GCN4-TRAK1$_{342-393}$	1	260	17.5	No binding		Fig. 2C
		2	260	17.5	No binding		
		3	260	17.5	No binding		
50 µM $CaCl_2$, 1 mM $MgCl_2$, 0.1 µM GTP	MIRO1$_{177-591}$ → MBP-TRAK1$_{342-431}$	1	465	40	1.11 ± 0.01	2.76 ± 0.17	Fig. 2D
		2	465	40	1.03 ± 0.02	5.38 ± 0.73	
		3	465	40	0.97 ± 0.03	5.95 ± 0.99	
5 (1) or 50 (2) µM $CaCl_2$, 1 mM $MgCl_2$, 0.1 µM GTP	$CaCl_2$ → MIRO1$_{177-591}$	1	800	105	No binding		Fig. 3A
		2	800	100	No binding		
5 mM to 0.08 µM EGTA, 1 mM $MgCl_2$, 0.1 µM GTP	$CaCl_2$ → MIRO1$_{177-591}$	1	800	97	2	K_{d1} = 0.30 ± 0.21<br>K_{d2} = 3.94 ± 2.92	Fig. 3B
		2	800	97	2	K_{d1} = 0.15 ± 0.08<br>K_{d2} = 6.80 ± 1.29	
		3	800	97	2	K_{d1} = 0.29 ± 0.16<br>K_{d2} = 7.58 ± 1.94	
5 mM EGTA, 1 mM $MgCl_2$, 0.1 µM GTP	MIRO1$_{177-591}$ → GCN4-TRAK1$_{342-431}$	1	320	14	1.84 ± 0.03	4.81 ± 0.44	Fig. 3C
		2	320	14	1.73 ± 0.04	3.52 ± 0.40	
		3	320	14	1.84 ± 0.03	5.13 ± 0.34	
5 mM EGTA, 1 mM $MgCl_2$, 0.1 µM GTP	MIRO1$_{177-591}$ → MBP-TRAK1$_{342-431}$	1	630	50	0.94 ± 0.002	6.02 ± 0.80	Fig. 3D
		2	630	55	0.97 ± 0.02	8.00 ± 0.94	
		3	630	55	1.09 ± 0.02	8.85 ± 1.09	
5 mM EGTA, 1 mM $MgCl_2$, 0.1 µM GTP	MBP-TRAK1$_{342-431}$ → MIRO1$_{177-591}$ E208A	1	460	30	0.90 ± 0.03	8.85 ± 1.10	Fig. 3E
		2	460	30	0.98 ± 0.03	9.09 ± 1.05	
		3	460	30	1.32 ± 0.02	3.86 ± 0.35	
5 mM EGTA, 1 mM $MgCl_2$, 0.1 µM GTP	MBP-TRAK1$_{342-431}$ → MIRO1$_{177-591}$ E328A	1	450	28	0.77 ± 0.05	9.17 ± 1.42	Fig. 3F
		2	450	28	0.80 ± 0.04	10.1 ± 1.17	
		3	450	28	0.82 ± 0.06	10.3 ± 1.60	
5 mM EGTA, 1 mM $MgCl_2$, 50 µM GDP	MBP-TRAK1$_{342-431}$ → MIRO1$_{177-591}$	1	585	48	0.80 ± 0.02	10.6 ± 1.18	Fig. 4D
		2	585	48	0.82 ± 0.02	24.8 ± 1.42	
		3	585	48	0.89 ± 0.07	20.9 ± 2.40	
5 mM EGTA, 1 mM $MgCl_2$, 50 µM GTP	MBP-TRAK1$_{342-431}$ → MIRO1$_{177-591}$	1	570	40	0.82 ± 0.04	8.93 ± 1.40	Fig. 4E
		2	570	40	0.83 ± 0.03	18.5 ± 1.27	
		3	570	40	0.99 ± 0.03	15.7 ± 1.19	
1 mM $MgCl_2$, 50 µM GTP	MBP-TRAK1$_{342-431}$ → MIRO1$_{410-591}$	1	400	35	No binding		Fig. 4F
		2	400	35	No binding		
		3	400	35	No binding		
5 mM EGTA, 1 mM $MgCl_2$, 0.1 µM GTP	MIRO1$_{177-591}$ → MBP-TRAK1$_{342-431}$ ^{400}KR^{401} to AA	1	370	35	0.85 ± 0.02	4.39 ± 0.40	Fig. 5B
		2	370	35	1.03 ± 0.02	2.98 ± 0.36	
		3	370	35	0.95 ± 0.03	3.98 ± 0.66	
5 mM EGTA, 1 mM $MgCl_2$, 0.1 µM GTP	MBP-TRAK1$_{342-431}$ ^{400}KR^{401} to AA → MIRO1$_{177-591}$	1	370	30	0.74 ± 0.04	7.52 ± 1.17	Fig. 5C
		2	370	30	0.58 ± 0.05	11.3 ± 1.78	
		3	370	30	0.63 ± 0.04	8.55 ± 1.30	
5 mM EGTA, 1 mM $MgCl_2$, 0.1 µM GTP	MIRO1$_{177-591}$ → MBP-TRAK1$_{342-431}$ ^{425}IPG^{427} to AAA	1	450	40	No binding		Fig. 5D
		2	450	40	No binding		
		3	450	40	No binding		
5 mM EGTA, 1 mM $MgCl_2$, 0.1 µM GTP	MBP-TRAK1$_{342-431}$ ^{425}IPG^{427} to AAA → MIRO1$_{177-591}$	1	410	35	No binding		Fig. 5E
		2	410	35	No binding		
		3	410	35	No binding		
5 mM EGTA, 1 mM $MgCl_2$, 0.1 µM GTP	TRAK1$_{394-434}$ → MIRO1$_{177-591}$	1	420	30	0.91 ± 0.03	9.62 ± 1.32	Fig. 6B
		2	420	25	0.78 ± 0.07	6.10 ± 1.49	
		3	420	30	0.90 ± 0.04	5.65 ± 1.13	
5 mM EGTA, 1 mM $MgCl_2$, 0.1 µM GTP	TRAK1$_{394-434}$ ^{425}IPG^{427} to AAA → MIRO1$_{177-591}$	1	700	50	No binding		Fig. 6C
		2	700	50	No binding		
		3	700	50	No binding		

Table S2. Primers used in this study

Construct	MW	Forward Primer (5' to 3')		Reverse Primer (5' to 3')		SDS-PAGE
MIRO1$_{1-591}$	67.7 kDa		TTCGAATTCATGAAGAAAGACGTGC GGATCCTG	Rev 1	AAACTGCGGGTGGCTCCAGAACGT GGAGCTCTTGAGGTCAGCTTG	Fig. 1B
				Rev 2	TTCGCGGCCGCTCACTTTTCAAACT GCGGGTGGCTCCAGAAC	
MBP-MIRO1$_{2-180}$	65.6 kDa		TTCGGTACCATGAAGAAAGACGTG CGGATCCTG		TTCGAATTCTCACTTTTCAAACTGC GGGTGGC	Fig. 1C
MIRO1$_{177-591}$	50.3 kDa		CTTGGATCCGAGGAGAAGGAGATG AAACCAGCTT		TTCGCGGCCGCTCACTTTTCAAACT GCGGGTGGCTCCAGAAC	Fig. 1D
MIRO1$_{177-591}$ E208A	50.3 kDa	QC	GTACTCTCAATGATGCTGCACTCAA CTTCTTTCAGAGG	QC	CCTCTGAAAGAAGTTGAGTGCAGC ATCATTGAGAGTAC	Fig. S5, Lane 1
MIRO1$_{177-591}$ E328A	50.3 kDa	QC	GAGACTGTGCTTTGTCACCTGATGC GCTTAAAGATTTATTTAAAG	QC	CTTTAAATAAATCTTTAAGCGCATCA GGTGACAAAGCACAGTCTC	Fig. S5, Lane 2
MBP-MIRO1$_{410-591}$	62.5 kDa		TTCGGATCCCAGAAAAAACAAACTC AAAGAAATGTGTTCAGATG		TTCGCGGCCGCTCACTTTTCAAACT GCGGGTGGCTCCAGAAC	Fig. S5, Lane 3
TRAK1$_{99-532}$	53.5 kDa		GGATCCTGCCGCAGAGAGGGTGG GACAGATGA	Rev 1	AAACTGCGGGTGGCTCCACTCTCC CTTCTCTGCCAGCTC	Fig. 1B-D
				Rev 2	TTCGCGGCCGCTCACTTTTCAAACT GCGGGTGGCTCCACTC	
GCN4-TRAK1$_{342-393}$	27.4 kDa (dimer)		TTCGAATTCATGAAACAGCTGGAGG ACAAAGTGGAAGAACTG	Rev 1	TTCCGGGTGGCTCCAGGACTCTGC CTCCTCCAGCTG	Fig. S5, Lane 4
GCN4-TRAK1$_{342-431}$	36.0 kDa (dimer)		TTCGAATTCATGAAACAGCTGGAGG ACAAAGTGGAAGAACTG	Rev 1	TTCCGGGTGGCTCCAGGACTCTGC CTCCTCCAGCTG	Fig. S5, Lane 5
				Rev 2	TTCGGTACCTCACTTTTCAAACTGC GGGTGGCTCCAGGACTCTG	
MBP-TRAK1$_{342-431}$	55.1 kDa (monomer)		TTCGGATCCGAGGCCCAGGAGGAG CTGAAG		TTCGGTACCTCACATGGCAGAGGA CTGATTGCTGC	Fig. S5, Lane 6
MBP-TRAK1$_{342-431}$ ^{400}KR^{401} to AA	55.0 kDa	QC 1	GACATCACACACCAGGCGAGGGTG TTCGAGACAGTGCGGAACATC	QC 1	GATGTTCCGCACTGTCTCGAACAC CCTCGCCTGGTGTGTGATGTC	Fig. S5, Lane 7
		QC 2	GACATCACACACCAGGCGGCGGTG TTCGAGACAGTGCGGAACATC	QC 2	GATGTTCCGCACTGTCTCGAACAC CGCCGCCTGGTGTGTGATGTC	
MBP-TRAK1$_{342-431}$ ^{425}IPG^{427} to AAA	55.1 kDa	QC 1	ACACCATCCCCCATGAACATCGCC GGCAGCAATCAGTCCTGGAGC	QC 1	GCTCCAGGACTGATTGCTGCCGGC GATGTTCATGGGGGATGGTGT	Fig. S5, Lane 8
		QC 2	ACACCATCCCCCATGAACATCGCC GCCAGCAATCAGTCCTGGAGC	QC 2	GCTCCAGGACTGATTGCTGGCGGC GATGTTCATGGGGGATGGTGT	
		QC 3	ACACCATCCCCCATGAACGCCGCC GCCAGCAATCAGTCCTGGAGC	QC 3	GCTCCAGGACTGATTGCTGGCGGC GGCGTTCATGGGGGATGGTGT	
TRAK1$_{394-434}$ WT and ^{425}IPG^{427} to AAA	4.8 kDa (peptide)		TTCGGATCCCCCGACATCACACAC CAGAAGAG	Rev 1	TTCGGTACCTCACATGGCAGAGGA CTGATTGCTGC	Fig. S5, Lanes 9 and 10
				Rev 2	TCACATGGCGGACTGATTGCTGGC GGCG	
				Rev 3	GGTACCTCACATGGCGGACTGATT GCTG	
